# Natural gum-based nanocomposite materials for the photocatalytic degradation of dye pollutants in wastewater: a review of recent advances, technology readiness levels, environmental threats, innovative pathways, and AI integration

**DOI:** 10.1039/d6ra06137k

**Published:** 2026-09-25

**Authors:** Stephen Sunday Emmanuel, Mustapha Omenesa Idris, Ademidun Adeola Adesibikan, Hamza Badamasi, Olumide James Oluwole

**Affiliations:** a Department of Industrial Chemistry, Faculty of Physical Sciences, University of Ilorin P. M. B. 1515 Ilorin Nigeria stephenemmanuel6011@gmail.com; b Interdisciplinary Research Center for Membranes and Water Security, King Fahd University of Petroleum and Minerals Dhahran 31261 Saudi Arabia; c Department of Chemistry, Federal University Dutse Dutse Jigawa State Nigeria

## Abstract

Photocatalytic degradation-oriented technology remains at the forefront of advanced oxidation processes for the removal of water pollutants like dyes. In the search for a cheap, green, eco-sustainable photocatalysts, natural gum-based nanomaterials (NGBNs) have garnered considerable attention. Thus, this review aims to present a critical analysis of the latest research findings and advances in the application of NGBNs for the photocatalytic degradation of dye pollutants in simulated wastewater. Notably, the study findings revealed that various NGBNs show a consistent trend of excellent degradation efficiency at a residence time of 1–760 minutes, a dose of 0.5–1000 mg, and a light intensity of 8–500 W. This remarkable efficiency was due to a narrow band gap and an abundance of oxygenated functional groups imparted to NGBNs by natural gums. Reusability and photostability analyses showed that NGBNs can be reused 3–7 times while still delivering ≥85% efficiency without seriously compromising the architectural morphology. Besides evaluating the efficiency of NGBNs for dye degradation, this review presents the ecotoxicity and ecological structure–activity relationships and identifies the strengths, weaknesses, opportunities, and threats that can serve as future directions to advance the research beyond grade 4 of the technology readiness level towards actual implementation in wastewater treatment systems.

## Abstract 2

Natural gum-based nanomaterials as green photocatalysts for dye degradation were reviewed, and the findings revealed that the presence of natural gum in the composite enhances ^·^OH/O_2_˙^−^ generation, e^−^/h^+^ separation/transfer, and improves performance.

## Graphical abstract

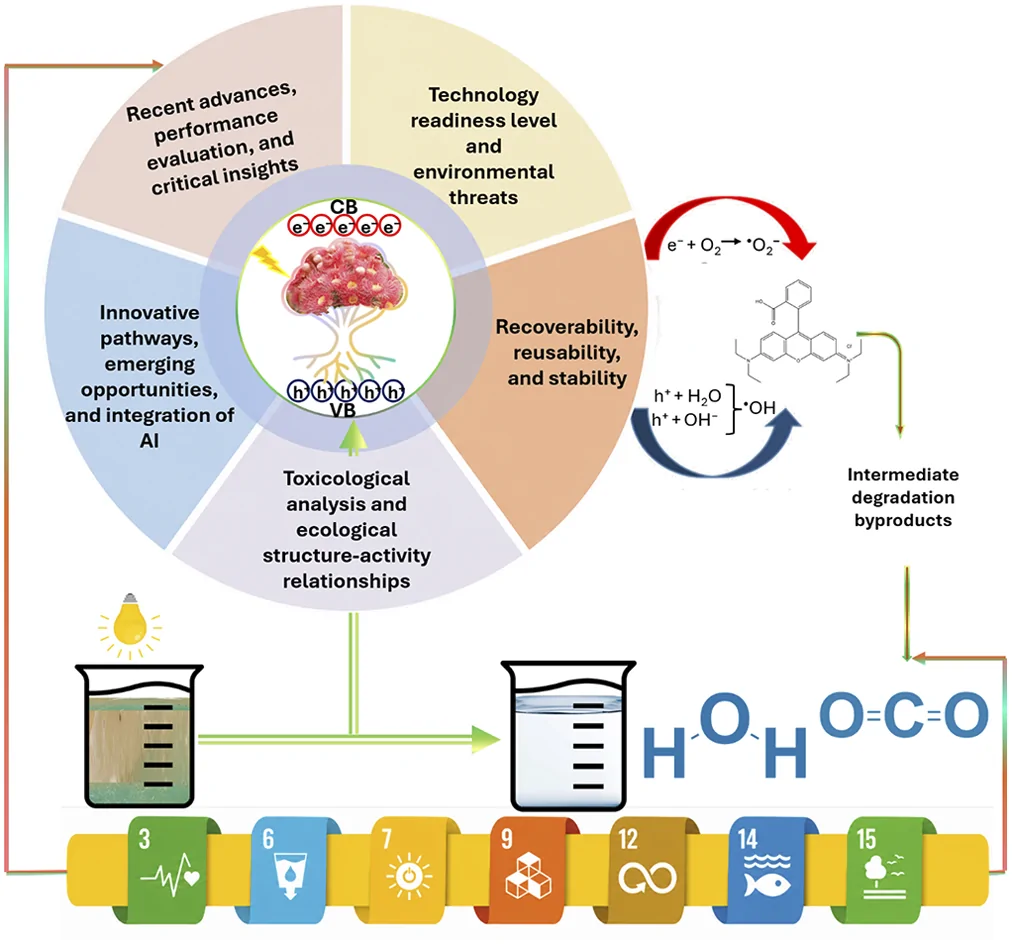

## 1. Introduction

Water is the mother of all natural resources, without which no human being can survive. Interestingly, in the 21st century, water has become blue gold owing to the consistent rise in water insecurity across the globe.^1,2^ This continuous upsurge in water insecurity (which is expected to affect 4.5 billion people by 2025) is largely due to anthropogenic activities, leading to the contamination of water bodies with various pollutants, including heavy metals, pesticides, pharmaceuticals, and dyes.^1,3,4^ Specifically, the contamination of water bodies with discharged dye effluents from textile, cosmetic, leather, pharmaceutical, and dyeing industries, *etc.*, poses dangerous ecological threats.^5,6^ Among these are degradation of water quality, impartation of unpleasant color/odour, and disruption of photosynthesis and aquatic life balance by blocking light penetration.^7–9^ Moreover, these dye pollutants are highly toxic, carcinogenic, and mutagenic even at low concentrations.^9,10^ Dyes are stubborn pollutants, chemically/thermally stable and resistant to biodegradation, which causes their accumulation in aquatic ecosystems. Consequently, they pose toxicity risks to fish, invertebrates, and other aquatic life, thereby causing biomagnification within the food chain/web.^9^ As summarized in Fig. 1, the long-term accumulation of dyes has been found to have far-reaching negative effects on health, economy, and the overall functioning of ecological systems.

**Fig. 1:**
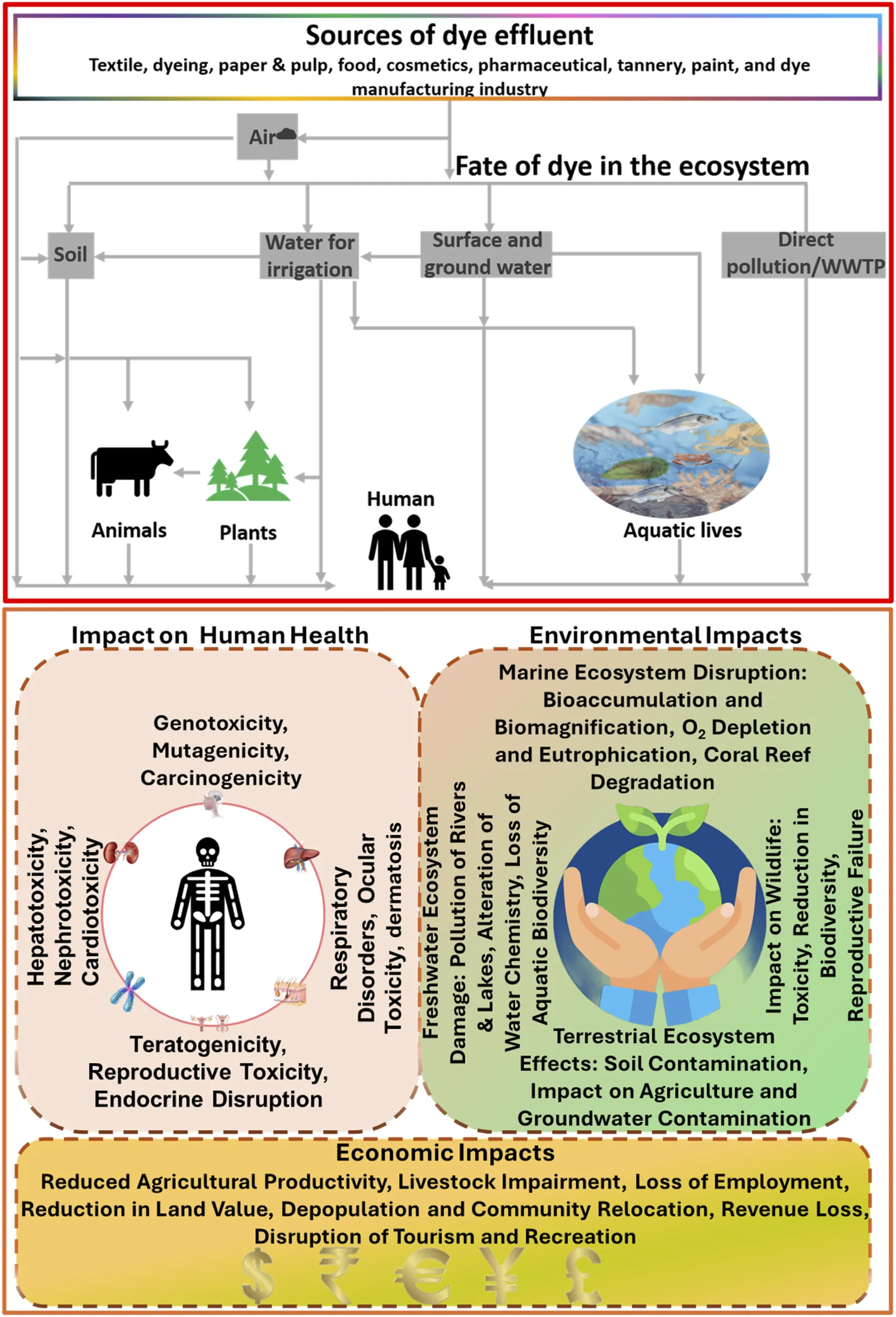
Overview of the fate of dye pollutants within the ecosystem and their negative impact.

Thus far, various conventional methods have been developed for the remediation of dye pollutants, including adsorption, flocculation/coagulation, the biological method, and electrolysis.^11,12^ However, these methods suffer from bottlenecks like incomplete degradation, generation of secondary pollutants, and often the transfer of the pollutant from one medium to another.^12,13^ Conversely, advanced oxidation processes (AOPs), particularly the photocatalysis technique, offer a promising alternative because of their ability to mineralize organic pollutants into harmless end-products, such as carbon dioxide and water, under light irradiation.^11,14–18^

In recent decades, the significant advancement of materials science and nanotechnology has promoted the development of various novel photocatalytic materials, including metal–organic frameworks,^19,20^ graphene/graphene oxide,^21,22^ MXenes,^23,24^ covalent organic frameworks,^25,26^ and nanocomposites.^27^ Nevertheless, as global interest in green chemistry principles and sustainable development goals grows, increasing emphasis is being placed on the environmental safety of the photocatalysts themselves.^28^ In that, beyond achieving high photocatalytic efficiency, it is equally important to curtail any secondary pollution or ecological risk associated with their application. Thus, this underscores the need to avoid or minimize toxic chemicals for the fabrication of photocatalyst materials.^29,30^ Moreover, photocatalysts are being upgraded from green single-function composites to multi-function composites for enhanced performance and recyclability. Consequently, in search of eco-sustainable photocatalysts, the development of nanocomposites from renewable, non-toxic, and low-cost natural materials has become popular within the research community.^28^ Within this context, the fabrication of natural gum-based nano-architecture materials has come to the limelight and is gradually garnering attention, as shown in Fig. 2. Natural gums, including xanthan gum, guar gum, and gum arabic, are green biomaterials that are abundant in nature.^28^ These natural gums have a plethora of inherent functional groups that can be imparted to the resulting green nanocomposite.^28,31^ Furthermore, among other unique advantages of NGBNs highlighted in the next section, the polymeric 3D networks of natural gums allow NGBNs to strongly combine with organic pollutants of interest to achieve a high photocatalytic degradation operation.^28,32,33^

**Fig. 2:**
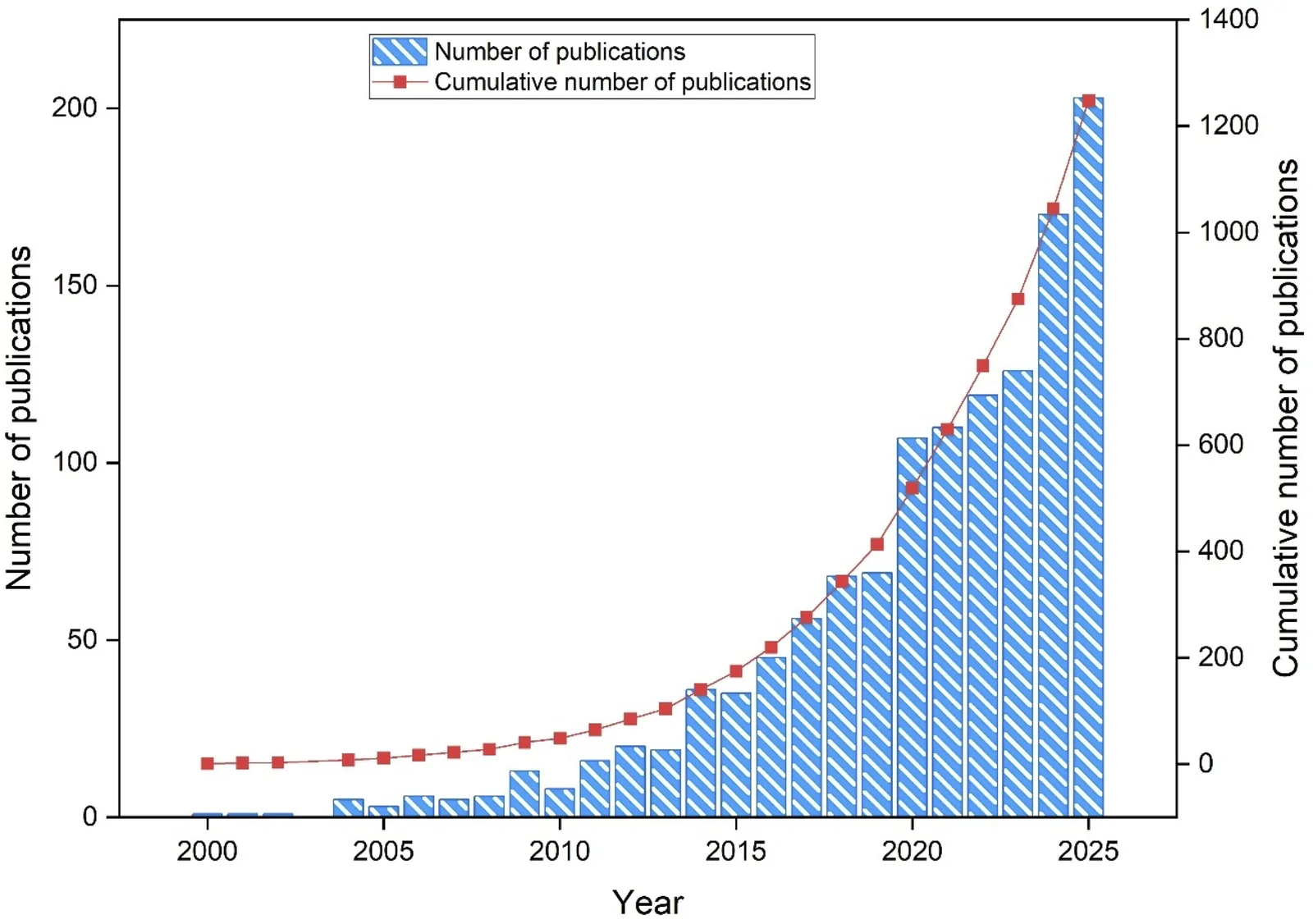
Publication trend/research growth on natural gum-based nano-architecture materials (from Scopus).

Notably, within the authors' extensive literature survey, there is no specific review solely directed toward the application of NGBNs as green nanocomposite photocatalysts for the degradation of hazardous dye pollutants. This is an important gap, and the novelty of this work lies in being the first review study on this subject. The core objectives of this review include a critical analysis of the latest research findings and advances, identifying trends, patterns, and parallels. Furthermore, the study pragmatically analyzed the photocatalytic performance, reusability, and photostability potential of NGBNs in order to shed light on their economic and industrial applicability values. Moreover, for the holistic evaluation of NGBNs' effectiveness and eco-friendliness, a toxicology study, as well as ecological structure–activity relationships, is presented. Furthermore, perspectives on the technology readiness level are succinctly discussed to evaluate the industrial applicability of NGBNs' photocatalytic system for dye degradation. Besides the aforementioned objectives, a SWOT (strengths, weaknesses, opportunities, and threats) analysis was conducted in this study in order to identify the challenges, dilemmas, and frontiers that can serve as future directions in the field alongside innovative pathways, emerging opportunities, and the potential of artificial intelligence integration.

## 2. Review methodology

A systematic literature search was conducted in international scientific repositories such as Scopus, Springer, Web of Science, Google Scholar, and Wiley & Sons to retrieve papers related to the study theme following the PRISMA guidelines (Preferred Reporting Items for Systematic Reviews and Meta-Analyses). The literature search was conducted using combinations of keywords, including “natural gum,” “natural gums,” “biopolymer,” “gum-based materials,” “gum nanocomposite,” “gum-based nanocomposite,” “biopolymer nanocomposite,” “natural polymer nanocomposite,” “green nanocomposite,” “photocatalytic degradation,” “photocatalysis,” “photocatalytic treatment,” “photocatalytic removal,” “dye degradation,” “dye removal,” “organic pollutant degradation,” “wastewater treatment,” “dye wastewater,” “water pollution,” “advanced oxidation processes,” and “semiconductor photocatalyst.” Additional searches were performed by combining these terms with specific natural gums, such as “gum arabic,” “guar gum,” “xanthan gum,” “tragacanth gum,” “acacia gum,” “gellan gum”, “bean gum”, “karaya gum,” and “tamarind gum,” as well as specific photocatalytic materials, including “TiO_2_,” “ZnO,” “CdS,” “g-C_3_N_4_,” “Fe_2_O_3_,” and “ZnS.” Specific dye pollutant names, including “methylene blue,” “methyl orange,” “Congo red,” “rhodamine B,” “malachite green,” “crystal violet,” and “reactive dyes,” were also used to identify studies investigating the photocatalytic degradation of individual dye pollutants using natural gum-based nanocomposite materials. Boolean operators (AND and OR) were used to combine these keywords and broaden or narrow the search results. Thereafter, all the co-authors manually screened the retrieved articles based on the titles to remove duplicate entries. Numerous publications were retrieved and then filtered as shown in Fig. 3 based on preset inclusion and exclusion criteria. First, we selected only peer-reviewed articles published in English and removed articles in other languages. Another criterion was screening the titles and abstracts and excluding opinions, encyclopedias, corrigenda, retracted articles, and seminar/conference abstracts. Moreover, publications where the full text was not accessible were excluded. Thereafter, publications that met the aforementioned criteria were considered suitable for the review study. In the end, a total of 47 articles were used to synthesize Table 1 and provide core analysis, while a total of 117 articles were finally employed for the overall synthesis of the review.

**Fig. 3:**
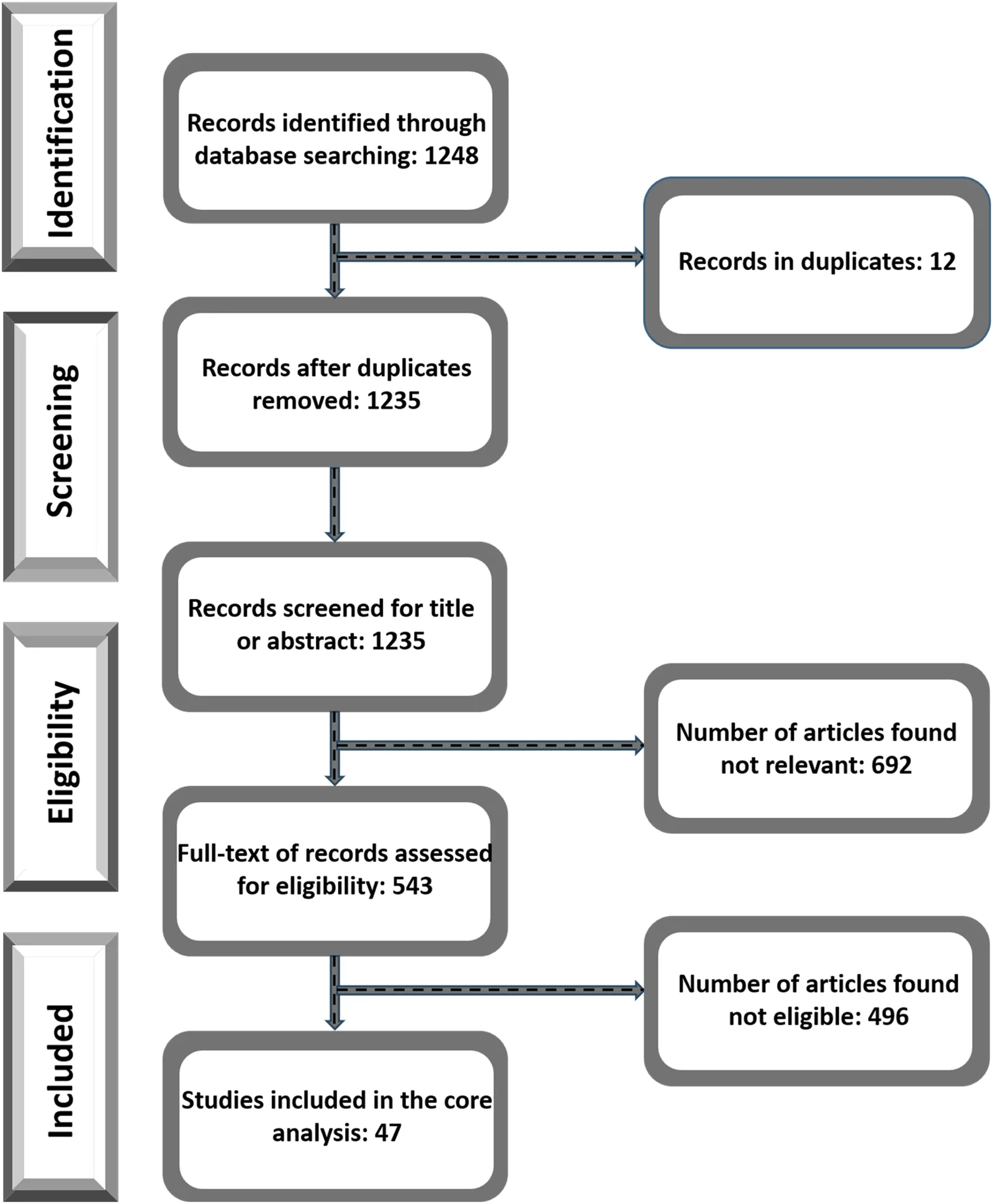
Schematic of the literature search and screening flow diagram. Adapted from ref. 34, which is open access and permits unrestricted use of materials under the terms of Creative Commons Attribution 4.0 Unported Licence.

**Table 1:** Summary of the performance of natural gum-based nanomaterials for the photocatalytic degradation of various dye pollutants^a^

Natural gum-based nanomaterial	Shape and IUPAC pore structure	Dye pollutant	Light source	Conditions/parameters	Time (min)	Degradation efficiency (%)	Ref.
Carboxymethyl tamarind kernel gum@TiO_2_	Spherical	Methylene blue	Sunlight	Dose = 20 mg, conc. = 200 mg L^−1^, pH = 9	120	96.80	67
Guar gum-agar@N–Zn hexacyanoferrate	NA	Indigo carmine	Sunlight	Conc. = 12 mg L^−1^, pH = 5, dose = 15	150	96	68
Guar gum-agar@N–Zn hexacyanoferrate	NA	Bismarck brown	Sunlight	Conc. = 20 mg L^−1^, pH = 9, dose = 20	180	91	68
Carrageenan-locust bean gum@Ag-TiO_2_	Mesoporous	Methylene blue	300 W xenon lamp	Dose = 500 mg, conc. = 40 mg L^−1^, pH = 7	80	96	60
Arabic gum@TiO_2_	Spherical mesoporous	Methylene blue	160 W mercury	Dose = 1000 mg L^−1^, conc. = 1.5 × 10^−5^ mol L^−1^	150	99	51
Karaya gum@TiO_2_	Spherical mesoporous	Methylene blue	160 W mercury lamp	Dose = 500 mg L^−1^, conc. = 12.0 × 10^−6^ mol L^−1^	150	90	52
Arabic gum@TiO_2_	Spherical mesoporous	Methylene blue	160 W mercury lamp	Dose = 500 mg L^−1^, conc. = 12.0 × 10^−6^ mol L^−1^	150	95	52
Xanthan gum@TiO_2_	Sheet-like	Methyl orange	Sunlight/H_2_O_2_	Dose = 40 mg, conc. = 30 mg L^−1^, pH = 5	120	89	64
Xanthan gum@TiO_2_	Sheet-like	Methyl orange	Sunlight	Dose = 40 mg, conc. = 30 mg L^−1^, pH = 5	120	74	64
Guar gum@CMC@SA@ZnO	NA	Reactive yellow	Sunlight	Dose = 30 mg, pH = 3	240	>94	58
Guar gum@CMC@SA@ZnO	NA	Reactive red	Sunlight	Dose = 30 mg, pH = 3	240	>94	58
Guar gum@CMC@SA@ZnO	NA	Reactive blue	Sunlight	Dose = 30 mg, pH = 3	240	>94	58
Guar gum@MgB_2_@Ru	Flower-like	Crystal violet	48 W 6 visible lamps	Dose = 7 mg, pH = ∼7, conc. = 7 mg L^−1^	100	99.7	55
Almond gum@BiO	Cluster mesoporous	Congo red	Xenon light	Dose = 100 mg, conc. = 15 mg L^−1^	135	90.21	38
Almond gum@BiO	Cluster mesoporous	Brilliant green	Xenon light	Dose = 100 mg, conc. = 15 mg L^−1^	120	90.52	38
Kondagogu gum@TiO_2_	Spherical	Methylene blue	Sunlight	Dose = 200 mg L^−1^, conc. = 10^−5^ M, pH = 7–9	90	96.85	59
Arabic gum@BaTiO_3_–Au–ZnO	Spherical	Rose bengal	350 W xenon lamp	Dose = 50 mg L^−1^, conc. = 15 mg L^−1^, pH = 7	90	94.7	35
Arabic gum@BaTiO_3_–Au–ZnO	Spherical	Brilliant green	350 W xenon lamp	Dose = 50 mg L^−1^, conc. = 15 mg L^−1^, pH = 10	90	88.31	35
Karaya gum@acrylamide@Ag	Spherical	Congo red	Visible light	Dose = 250 mg L^−1^	90	73.25	69
Gum arabic@CuFe_2_O_4_–Ag	Spherical	Methylene blue	UV-vis	Dose = 1 mg mL^−1^, conc. = 30 mg L^−1^	150	90	70
Gum acacia@TiO_2_	NA	Rhodamine-B	TUV LED tube Philips lighting	Dose = 30 mg, conc. = 10 mg L^−1^, pH = 2–12	30	100	71
Gum acacia@TiO_2_	NA	Methyl orange	TUV LED tube Philips lighting	Dose = 30 mg, pH = 2, conc. = 10 mg L^−1^	30	95	71
Guar gum@Fe_3_O_4_	Octahedron with circle and square	Crystal violet	Sunlight	Dose = 800 mg L^−1^, pH = 6, conc. = 15 mg L^−1^	100	98.94	61
Guar gum@Fe_3_O_4_	Octahedron with circle and square	Indigo carmine	Sunlight	Dose = 800 mg L^−1^, pH = 6, conc. = 15 mg L^−1^	120	97.61	61
Gum arabic/guar gum@CuO	Irregular and rough	Methylene blue	Sunlight	Dose = 100 mg, conc. = 10 mg L^−1^	80	>80	32
Gum arabic/guar gum@ZnO	Irregular and rough	Methylene blue	Sunlight	Dose = 100 mg, conc. = 10 mg L^−1^	80	>80	32
Gum arabic/guar gum@Fe_3_O_4_	Irregular and rough	Crystal violet	Sunlight	Dose = 100 mg, conc. = 10 mg L^−1^	80	>80	32
Gum arabic/guar gum@ZnO	Irregular and rough	Crystal violet	Sunlight	Dose = 100 mg, conc. = 10 mg L^−1^	80	>80	32
Gum arabic/guar gum@Fe_3_O_4_	Irregular and rough	Crystal violet	Sunlight	Dose = 100 mg, conc. = 10 mg L^−1^	80	>80	32
Guar gum/Al_2_O_3_	Bean-like	Malachite green	Sunlight	Dose = 100 mg, conc. 1.5 × 10^−5^ M, pH = 7	220	90	62
Xanthan gum@TiO_2_	Cluster and irregular	Reactive red	Sunlight/H_2_O_2_	Dose = 25 mg L^−1^, pH = 5, conc. = 25 mg L^−1^	120	92.5	65
Xanthan gum@TiO_2_	Cluster and irregular	Reactive turquoise	Sunlight/H_2_O_2_	Dose = 25 mg L^−1^, pH = 5, conc. = 25 mg L^−1^	120	90.8	65
Arabic gum@ZnO	Spherical	Direct blue 129	80 W fluorescent lamp	Dose = 500 mg, conc. 20 mg L^−1^	105	95	72
Guar gum-crosslinked soya lecithin@ZVI	Rod-like	Methyl violet	Sunlight/H_2_O_2_	Dose = 30 mg, conc. 30 mg L^−1^, pH = 7	120	81	37
Tragacanth gum@TiO_2_	Sphere/sheet-like mesoporous	Crystal violet	8 W 4 TUV Philips lamps	Dose = 5 mg, conc. = 5 mg L^−1^, pH = 6	150	85	73
Agar gum@Ag@cellulose	Spherical	Malachite green	100 W halogen lamp	Dose = 20 mg, pH = 9, conc. = 15 mg L^−1^	60	98.92	74
Fenugreek gum@DDM@TiO_2_	Spherical	Congo red	Xe lamp	Dose = 250 mg L^−1^	75	95.27	75
Fenugreek gum@DDM@TiO_2_	Spherical	Methylene blue	Xenon lamp	Dose = 250 mg L^−1^	150	73.26	75
Karaya gum@g-C_3_N_4_@ZnO@SiO_2_	Rod/spherical-like	Trypan blue	350 W xenon lamp	Dose = 350 mg, conc. = 20 mg L^−1^, pH = 3	90	92.22	76
Karaya gum@g-C_3_N_4_@ZnO@SiO_2_	Rod/spherical-like	Congo red	350 W xenon lamp	Dose = 350 mg, conc. = 20 mg L^−1^, pH = 3	90	89.70	76
Karaya gum@g-C_3_N_4_@ZnO	Rod/spherical-like	Trypan blue	350 W xenon lamp	Dose = 350 mg, conc. = 20 mg L^−1^, pH = 3	90	78.74	76
Karaya gum@g-C_3_N_4_@ZnO	Rod/spherical-like	Congo red	350 W xenon lamp	Dose = 350 mg, conc. = 20 mg L^−1^, pH = 3	90	73.58	76
Guggul gum@BiFeO_3_	Spherical	Magenta-O	Sunlight	Dose = 500 mg, conc. = 10 mg L^−1^	760	98.8	63
Guar gum-agar agar@N-doped ZnO	Spherical	Yellow dye	Sunlight	Dose = 20 mg, pH = 7, conc. = 2 mg L^−1^	150	92	33
Guar gum@polyacrylic acid@TiO_2_	Spherical	Methylene blue	UVA–UVB Philips lamps	Dose = 500 mg, conc. 20 mg L^−1^, pH = 8	NA	>95	77
Guar gum@carboxymethyl cellulose@ZnO–Fe_2_O_3_	Flake/sheet-like	Crystal violet	Sunlight	Dose = 20 mg, conc. = 5 mg L^−1^, pH = 7	120	92	31
Guar gum@carboxymethyl cellulose@ZnO–Fe_2_O_3_	Flake/sheet-like	Methylene blue	Sunlight	Dose = 5 mg, conc. = 3 mg L^−1^, pH = 7	120	95	31
Guar gum@MnO_2_	NA	Reactive yellow	8 W blue light bulb	Dose = 2250 mg L^−1^, conc. = 10 mg L^−1^, pH = 3	30	81	78
Tragacanth gum@CuO	NA	Safranin	Sunlight	Conc. 10 mg L^−1^, dose = 10 mg	60	∼100	79
Gum acacia@ZnO	Hexagonal/spherical	Methyl green	UV light	Conc. = 10^−5^ M	60	95	80
Guar gum@Ag@MIL-100(Fe)	Octahedral	Methylene blue	500 W xenon lamp	Conc. = 40 mg L^−1^	100	∼100	41
Gum tragacanth@TiO_2_	NA	Methylene blue	9 W UV Philips lamp	Dose = 150 mg L^−1^, pH = 11, conc. = 10 mg L^−1^	180	87	81
Gellan gum@TiO_2_	Granules mesoporous	Methylene blue	125 W tungsten lamp	Conc. = 1.2 × 10^−5^ mol L^−1^, dose = 500 mg L^−1^	120	95	82
Xanthan gum@agar@ZnO	Leaf-like	Malachite green	300 W xenon lamp	pH = 8, conc. = 70 mg L^−1^	46	99.45	57
Gellan gum@TiO_2_	Irregular macroporous	Methyl orange	UV light	NA	180	88.24	83
Xanthan gum@Cu-Fe_3_O_4_	Ellipsoidal/spherical	Methylene blue	Tungsten lamp	Dose = 0.5 mg, conc. = 1 mM	3.33	98	66
Xanthan gum@Cu-Fe_3_O_4_	Ellipsoidal/spherical	Crystal violet	Tungsten lamp	Dose = 0.5 mg, conc. = 1 mM	1.0	90	66
Xanthan gum@Cu-Fe_3_O_4_	Ellipsoidal/spherical	Congo red	Tungsten lamp	Dose = 0.5 mg, conc. = 1 mM	10.17	98	66
Xanthan gum@Cu-Fe_3_O_4_	Ellipsoidal/spherical	Methyl orange	Tungsten lamp	Dose = 0.5 mg, conc. = 1 mM	7.17	92	66
Gum acacia-activated carbon@CaO/NiO	Spherical	Methylene blue	365 W UV table lamp	Dose = 20 mg, conc. = 25 mg L, pH = 4	80	98.6	84
Gum acacia-activated carbon@CaO/NiO	Spherical	Methyl orange	365 W UV table lamp	Dose = 20 mg, conc. = 25 mg L, pH = 4	100	95.8	84
Gum acacia-activated carbon@CaO/NiO	Spherical	Methyl red	365 W UV table lamp	Dose = 20 mg, conc. = 25 mg L, pH = 4	100	92.4	84
Gum acacia-activated carbon@CaO/NiO	Spherical	Rhodamine B	365 W UV table lamp	Dose = 20 mg, conc. = 25 mg L, pH = 4	75	94.6	84
Gum acacia-activated carbon@CaO/NiO	Spherical	Methylene blue	Sunlight	Dose = 20 mg, conc. = 25 mg L, pH = 6	120	99.7	84
Gum acacia-activated carbon@CaO/NiO	Spherical	Methyl orange	Sunlight	Dose = 20 mg, conc. = 25 mg L, pH = 4	100	98.3	84
Gum acacia-activated carbon@CaO/NiO	Spherical	Methyl red	Sunlight	Dose = 20 mg, conc. = 25 mg L, pH = 4	100	96.7	84
Gum acacia-activated carbon@CaO/NiO	Spherical	Rhodamine B	Sunlight	Dose = 20 mg, conc. = 25 mg L, pH = 4	75	93.5	84
Gum acacia-crosslinked-poly(acrylamide) hydrogel@C_3_N_4_/BiOI	Circular/sheet	Crystal violet	Sunlight	Dose = 20 mg, conc. = 30 mg L^−1^, pH = 9	180	88	53
Locust bean gum@Zr(iv) selenophosphate	Mesoporous	Fast sulphon black	Sunlight	Dose = 150 mg, pH = 3, conc. = 1 × 10^−5^ M	180	90	85
Locust bean gum@Zr(iv) selenophosphate	Mesoporous	Crystal violet	Sunlight	Dose = 150 mg, pH = 9, conc. = 1 × 10^−5^ M	180	91.4	85
Bael gum-ZnO/Ag_2_CO_3_	Polyhedral/rod-like/spherical	Amido black 10B	350 W xenon lamp	Dose = 40 mg, conc. = 15 mg L^−1^, pH = 7	120	91.03	56
	Mesoporous						
Gaur gum@CuO	Spherical	Malachite green	Sunlight	Dose = 100 mg, conc. 1.5 × 10^−5^ M	120	89	86
Gum arabic-crosslinked-poly(acrylamide)/Ni(OH)_2_/FeOOH	Globular	Methylene blue	Sunlight	Conc. 50 mg L^−1^, dose = 20 mg, pH = 7	120	75	87
Tragacanth gum@TiO_2_	NA	Methylene blue	9 W Philips UV lamp	Dose = 130, conc. = 9.37 mg L^−1^, pH = 9.02	124.34	88.86	88
Tragacanth gum@CeO_2_@rGO	NA	Methylene blue	Sunlight	Dose = 2 mg, conc. 20 mg L^−1^	300	∼91	89
Poly(vinyl alcohol)-tragacanth gum@TiO_2_	Cauliflower/cube-like mesoporous	Malachite green	8 W Philips TUV lamp	Dose = 80 mg, conc. = 10 mg L^−1^, pH = 6	165	94.57	50
Xanthan gum@TiO_2_	NA	Methylene blue	36 W Osram UVC lamp	Conc. = 5.0 mg L^−1^, pH = 7	312	95	90

a NA = not available/not reported.

## 3. Unique advantages of natural gum-based nanomaterials

Notably, as presented in Fig. 4, natural gum-based nanomaterials as green photocatalysts have quite a number of advantages over their counterpart without natural gum. First, NGBNs have the ability to generate more radicals than their counterparts owing to the presence of abundant oxygenated functional groups like OH, COOH, and OCH_3_, endowed by natural gum.^28,31^ More specifically, numerous hydrophilic terminal groups (–OH and –O) on the NGBN surface can boost their interaction with other nanosemiconductors as well as with H_2_O molecules or target organic pollutants, both of which are essential for the mobility of e^−^ inside the nanocomposite and the chemical reaction in aqueous solutions.

**Fig. 4:**
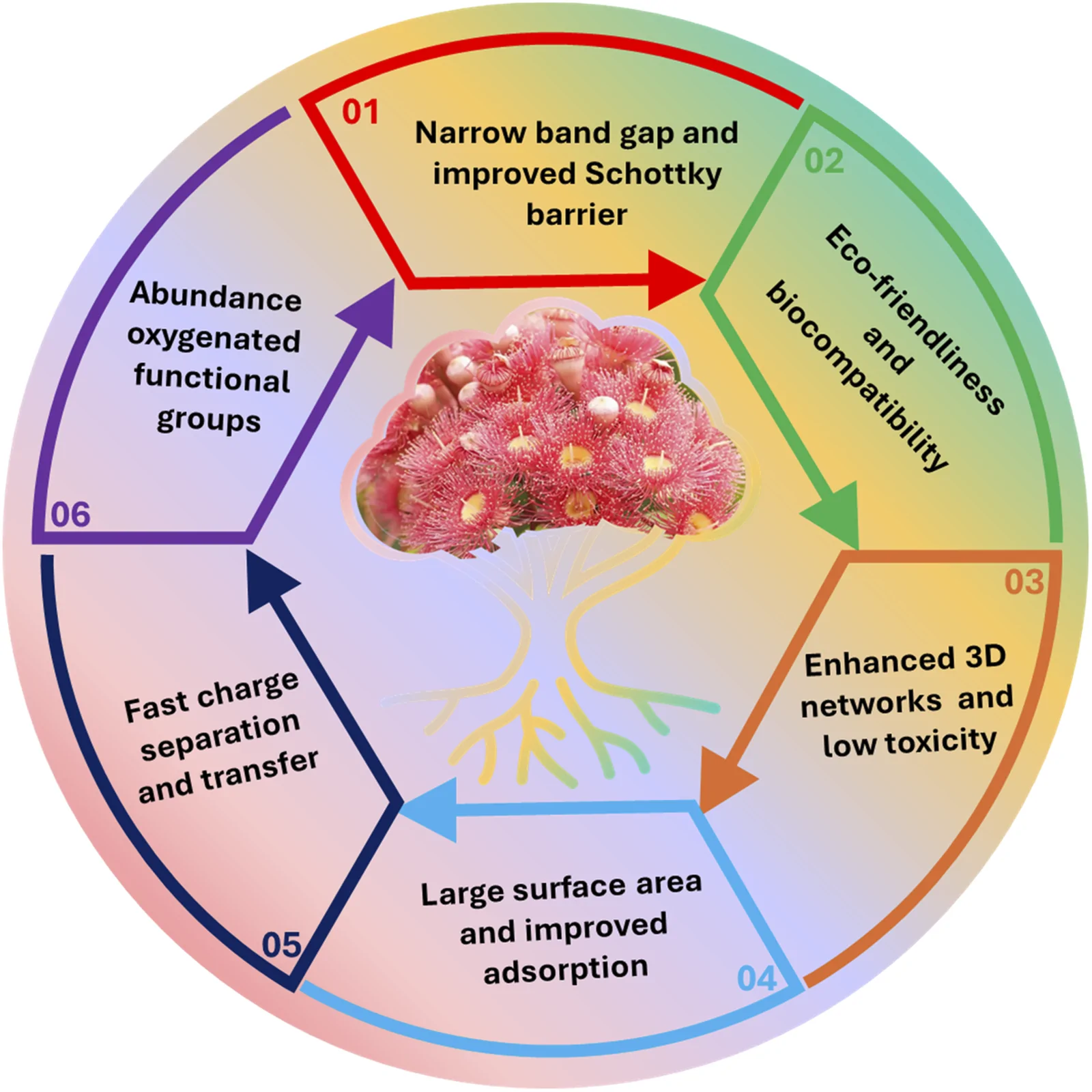
Unique advantages of NGBNs.

Furthermore, given the improved large surface area (SA) of NGBNs,^33,35^ the natural gum present in NGBNs has the ability to serve as a good support for other nanophotocatalysts such as CuO, ZnO, and TiO_2_ to prevent or reduce agglomeration and enhance the nanocomposite performance.^36,37^ This improved SA, coupled with the presence of oxygenated functional groups, then enhances dye adsorption and strengthens their interaction with active photocatalytic sites.^28,35^ Moreover, it has been reported that natural gum can act not only as a robust support but also as a starting material for the *in situ* engineering of metal oxide photocatalysts. The natural gum polymeric 3D network prevents the nanoparticles' ionic structures from degrading, thereby minimizing leaching and toxicity.^28,32,33^ Another uniqueness of NGBNs lies in the establishment of a Schottky junction to enhance the photo-generated charge carrier separation. Moreover, the creation of a Schottky barrier prevents photo-generated e^−^ from instantaneously accessing the NPs/semiconductor surfaces of the NGBM, hence reducing the rate of e^−^/h^+^ pair recombination while promoting photocatalytic activities.^35^ These unique advantages play a crucial role in the photocatalytic degradation of dye pollutants by NGBNs, as discussed in the next section.

## 4. Fundamental photocatalytic degradation mechanism

Photocatalytic degradation is a light-driven process in which a nanocatalyst, such as NGBNs, harnesses photon energy to transform organic contaminants, including dye pollutants, into harmless end-products like CO_2_, H_2_O, and mineralized ions (Fig. 5).^30,38^ Upon irradiation with light of energy equal to or exceeding the NGBN's band gap, photons are absorbed, and then e^−^ are photochemically generated at the surface of the NGBN.^39–42^ Afterward, e^−^ from the valence band (VB) is excited to the conduction band (CB), in tandem generating a hole known as h^+^ in the VB.^18,30,41,43^ The NGBN photocatalyst helps reduce the recombination of the h^+^ and e^−^ by stimulating electrons at higher energy levels.^44^ These photogenerated charge carriers trigger redox reactions. Specifically, CB electrons reduce dissolved molecular O_2_ to superoxide radicals (˙O_2_^−^), whereas h^+^ in the VB oxidize water or hydroxide ions to hydroxyl radicals (˙OH).^45–49^ As illustrated in Fig. 5, the resulting ˙OH and ˙O_2_^−^ attack the dye pollutant molecules at vulnerable C- or N-centered positions, initiating fragmentation through pathways such as demethylation, deamination, aromatic ring opening, decarboxylation, azo bond breakage, dealkylation, and C–N/S–N bond cleavage.^37,50^ These transformations ultimately yield low-molecular-weight intermediates (*e.g.*, HCOOH and CH_3_COOH) that are further mineralized to CO_2_, H_2_O, NH_4_^+^, *etc.*^37,50^

**Fig. 5:**
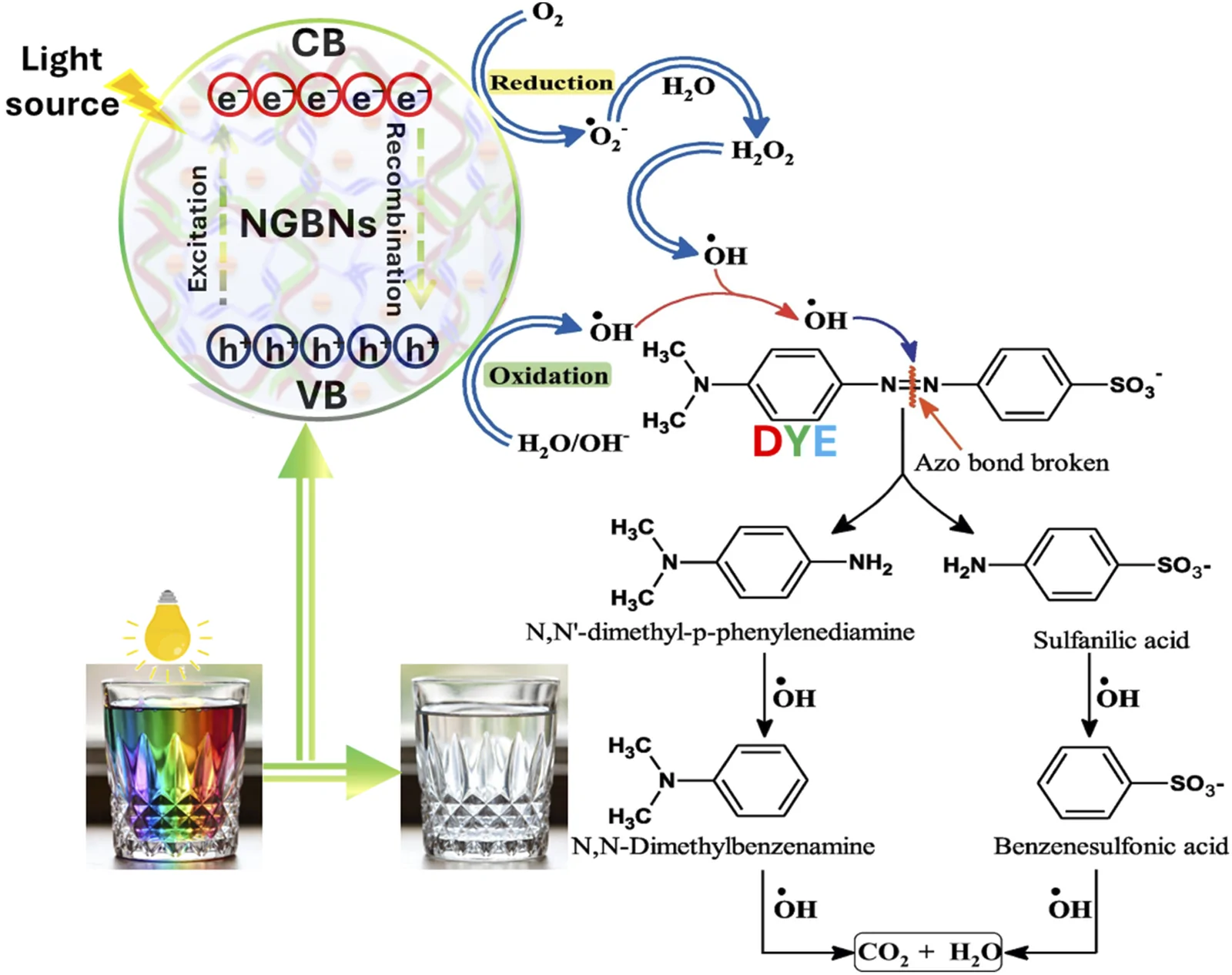
Unified schematic of the photocatalytic degradation mechanism. Adapted with permission from ref. 12. Copyright, John Wiley & Sons Ltd, 2023.

Although studies consistently identify ˙O_2_^−^ and ˙OH as the dominant reactive oxygen species responsible for dye pollutant degradation, direct participation of photogenerated e^−^/h^+^ pairs has also been reported in certain systems. Chromatographic and spectroscopic monitoring reveals that the characteristic absorption peaks of the parent dye molecule initially appear during the early stages of radical attack, reflecting transient modifications *via* the aforementioned pathways.^37,50^ As the reaction progresses, these parental signals progressively diminish and are succeeded by new peaks corresponding to fragmented intermediates and final mineralization products, confirming the sequential breakdown and ultimate detoxification of the target dye pollutants.^37,38,50^

## 5. Recent advances and critical insights into the degradation performance of various natural gum-based nanomaterials

In this section, notable research works that have been executed on the applications of various natural gum-based nano-architectural materials for the photocatalytic degradation of various dye pollutants were pragmatically discussed on a case-by-case basis while highlighting the recent advancements. For instance, in a particular case study,^51^ arabic gum@TiO_2_ achieved an MB degradation efficiency of 99% within 150 min under 160 W mercury-lamp irradiation at a maximum catalyst dose of 1000 mg L^−1^. The study attributed the enhanced degradation primarily to the interaction of the NGBN with the incident light and the subsequent generation of reactive species capable of attacking MB. Interestingly, findings revealed that increasing the photocatalyst dosage does not necessarily result in proportional improvement in degradation efficiency. Excessive NGBN loading may promote particle aggregation, increase light scattering and shielding, and reduce the effective penetration of photons through the reaction medium. Furthermore, excessive adsorption of MB and its transformation products on the catalyst surface may progressively block active sites and hinder interaction between the pollutant and photogenerated reactive species.^51^ Therefore, from a critical perspective, the reported performance should be interpreted in the context of the experimental conditions, particularly the relatively high catalyst dosage and the specific irradiation source, which may limit direct comparison with studies conducted under different pollutant concentrations, catalyst loadings, and light intensities. These observations highlight the importance of optimizing the pollutant-to-catalyst ratio rather than assuming that the higher catalyst loading invariably improves the photocatalytic performance.

The enhanced photocatalytic activity of gum-modified TiO_2_ has also been associated with changes in its optical and interfacial properties. For example, karaya gum@TiO_2_ and arabic gum@TiO_2_ achieved more than 90% MB degradation after 150 min of mercury-lamp irradiation, compared with less than 25% degradation in the absence of the natural gum-based materials.^52^ This is in very good agreement with what was achieved by gum acacia-crosslinked-poly(acrylamide) hydrogel@C_3_N_4_/BiOI for the photocatalytic degradation of crystal violet.^53^ The authors attributed this enhancement to the lower reported band-gap values of NGBN, compared with pristine components, suggesting improved utilization of the incident irradiation and potentially more favourable photogenerated charge-carrier processes through heterojunction formation, like the direct Z-scheme reported for gum acacia-crosslinked-poly(acrylamide) hydrogel@C_3_N_4_/BiOI, as shown in Fig. 6.^53^ The major advantages of this NGBN direct Z-scheme are that it eliminates the need for an electronic mediator, enables retention of strong redox potentials, and allows charge carriers to migrate more efficiently than in type-II heterojunctions. This arises because the electrostatic attractive force of electron–hole pairs may lower the recombination barrier, whereas electron transfer between the CBs of type-II heterojunctions requires overcoming the Coulomb repulsion between electrons.^53,54^ In addition, at the molecular level, the hydroxyl, carboxyl, and other oxygen-containing functional groups present in natural gums can interact with metal precursors and metal oxide NP surfaces through coordination, H-bonding, or other interfacial interactions. Such interactions may influence nanoparticle nucleation and dispersion, suppress aggregation, and modify the surface environment of the semiconductor, thereby contributing to changes in light absorption and interfacial charge-transfer behaviour. Nevertheless, the specific contribution of these interactions should be distinguished from the intrinsic photocatalytic properties of the semiconductor, as direct molecular-level evidence is not consistently reported across NGBN systems.

**Fig. 6:**
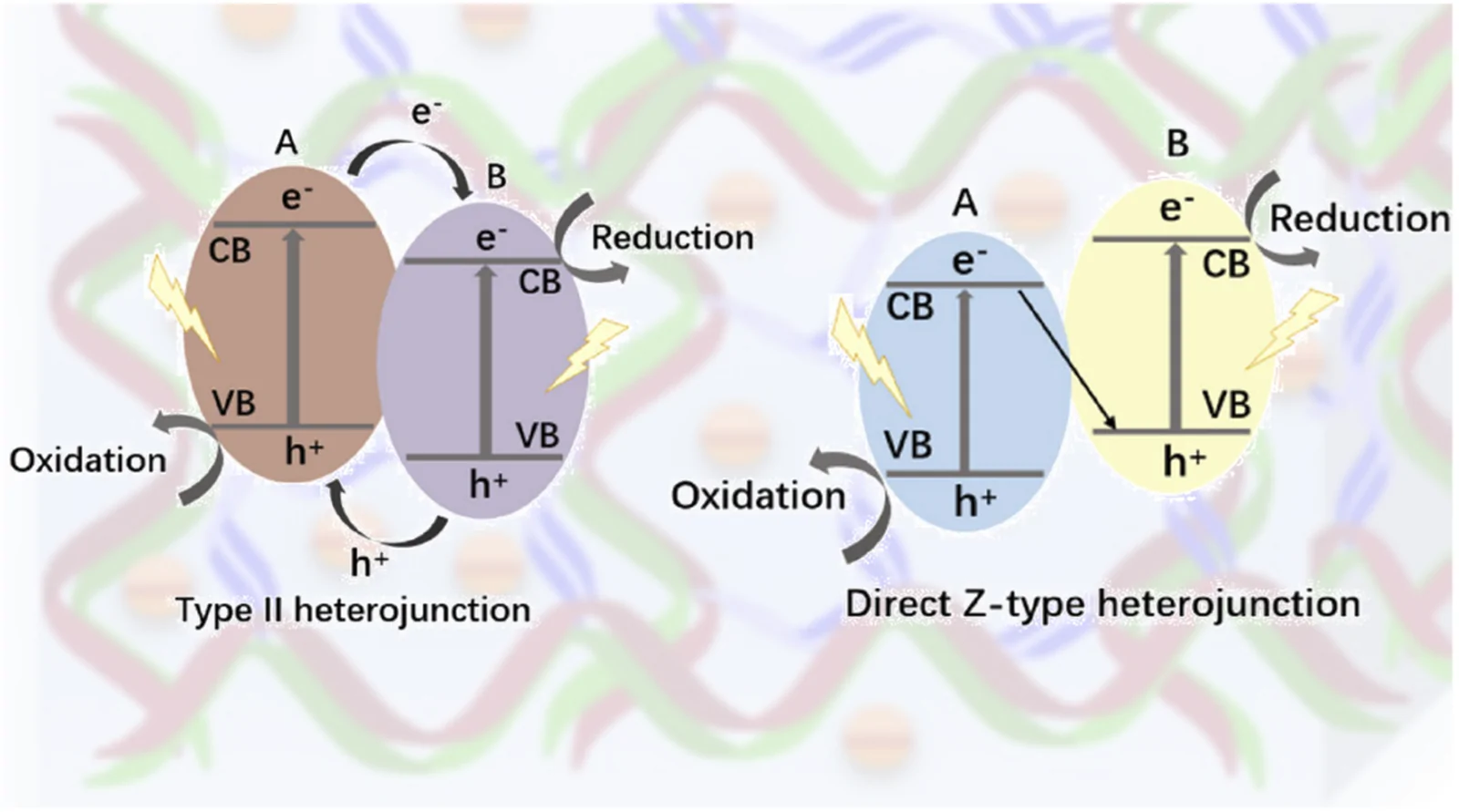
Typical type-II and direct Z-scheme heterojunctions for the NGBN photocatalytic mechanism. Adapted with permission from ref. 54. Copyright, John Wiley & Sons Ltd, 2026.

In a recent study, the incorporation of arabic gum into BaTiO_3_–Au–ZnO and the use of guar gum in MgB_2_–Ru resulted in substantially higher dye removal than the corresponding inorganic systems, with rose bengal degradation increasing from 38.20% for BaTiO_3_ to 94.7% for arabic gum@BaTiO_3_–Au–ZnO and crystal violet degradation approaching 100% for guar gum@MgB_2_–Ru compared with 53.7% for MgB_2_.^35,55^ These findings suggest that the gum component can promote the accumulation of pollutant molecules at or near photocatalytically active sites through enhanced adsorption. At the molecular level, O-containing groups within polysaccharide matrices of the gum participate in various interactions (H-bonding, electrostatic interactions, and coordination or surface complexation) with exposed metal centres, thereby modifying the interfacial environment of the nanoparticles. The importance of these enhanced surface interactions by the NGBN functional groups is particularly evident when the degradation values are considered alongside the role of adsorption. The high removal obtained with guar gum@MgB_2_–Ru, for instance, was partly attributed to the adsorption of crystal violet during the dark period, suggesting that the gum-derived carbon networks and functional groups act not merely as a passive support but as an interfacial component that concentrates the pollutant around the photocatalytically active phase.^55^ A similar contribution of surface interactions can be inferred for bael gum-ZnO/Ag_2_CO_3_, where gum-associated hydroxyl groups were proposed to strengthen interactions with Amido black 10B.^56^ Aside from the bael gum acting as a stabilizing agent, the NGBN forms an n–p heterojunction (bael gum-ZnO (n)/Ag_2_CO_3_ (p)). A unique advantage of this n–p heterojunction is that the built-in electric field at the n–p interface drives photogenerated charge carriers in opposite directions, as shown in Fig. 7, promoting efficient charge separation and photocatalytic degradation.^56^ However, when compared to other studies analysed in the previous paragraphs, this also introduces an important methodological consideration when comparing the reported degradation efficiencies. The variation in degradation efficiency among NGBNs may also arise from differences in the heterojunction architectures employed, as distinct interfacial charge-transfer pathways can result in different efficiencies of charge separation, carrier migration, and suppression of electron–hole recombination. Thus, NGBNs with different heterojunction configurations may exhibit substantially different photocatalytic degradation efficiencies even under otherwise similar conditions. Therefore, studies should ideally report and systematically compare the specific heterojunction architectures employed, together with their corresponding charge-transfer and separation characteristics, to clarify their contribution to variations in photocatalytic degradation efficiency. Without such information, the substantially different degradation percentages reported for gum-based nanocomposites cannot be attributed solely to the intrinsic effect of the gum or to improved photocatalytic kinetics.

**Fig. 7:**
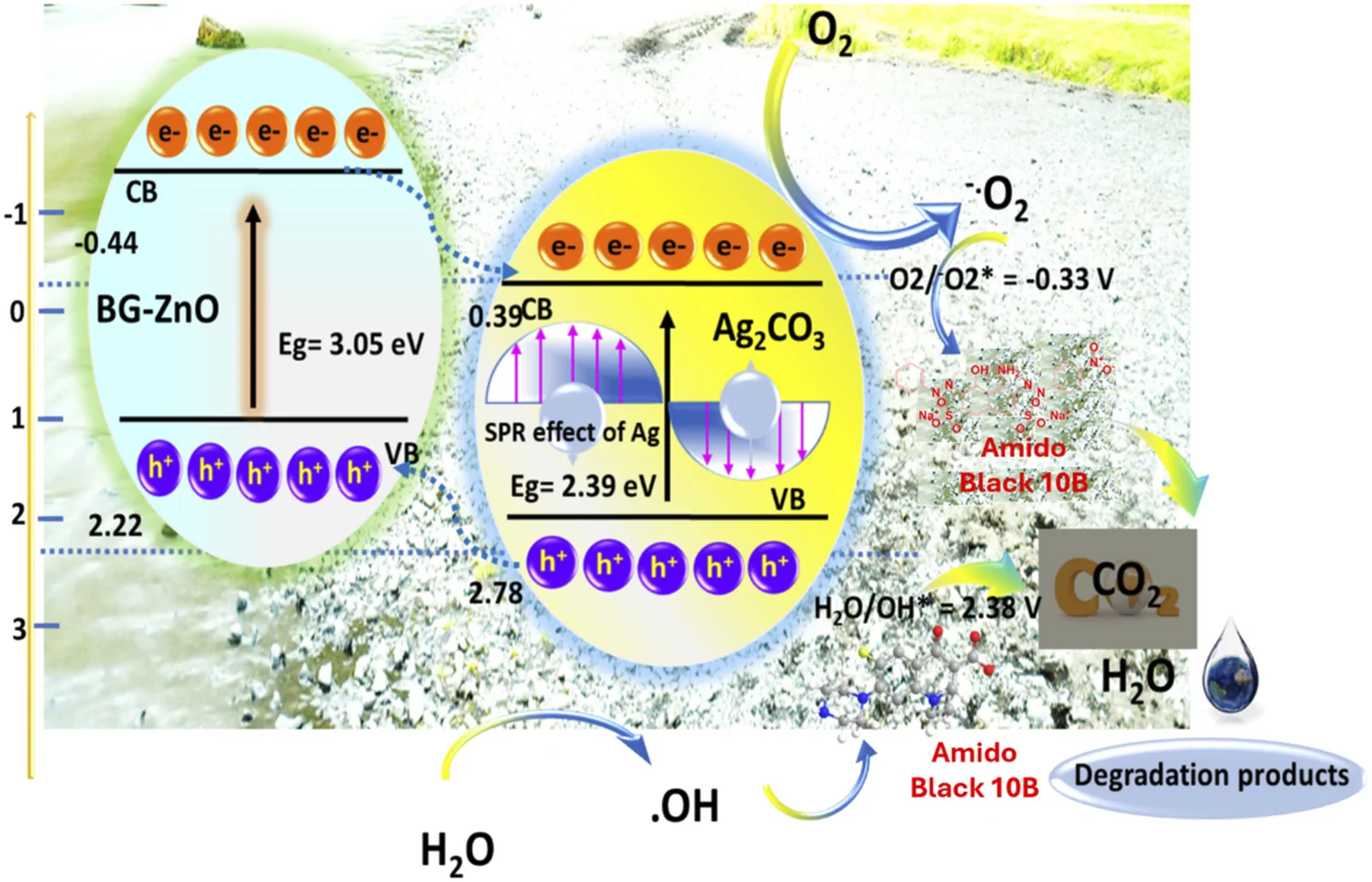
Proposed n–p heterojunction mechanism of bael gum-ZnO/Ag_2_CO_3_ for the photocatalytic degradation of Amido black 10B under visible light irradiation. Reproduced with permission from ref. 56. Copyright, Elsevier B.V., 2025.

Beyond surface adsorption, the gum matrix has been demonstrated to influence photocatalytic performance through the modification of the electronic and interfacial properties of the inorganic phase. The remarkably higher degradation obtained for arabic gum@BaTiO_3_–Au–ZnO relative to BaTiO_3_ and BaTiO_3_–ZnO illustrates the potential contribution of multicomponent heterostructure formation. Specifically, the incorporation of ZnO and Au was associated with broader light absorption, modified band-gap characteristics, enhanced charge transport, and improved e^−^/h^+^ separation.^35^ Mechanistically, as shown in Fig. 8, the Au component additionally functions as an electron sink through the formation of a Schottky barrier at the Au–semiconductor interface, reducing the probability of rapid e^−^/h^+^ recombination and thereby increasing the lifetime of charge carriers available for reactive oxygen species generation. Natural gums may further influence these processes through their interaction with nanoparticle surfaces and metal centres, potentially altering surface electronic states and facilitating interfacial charge transfer.

**Fig. 8:**
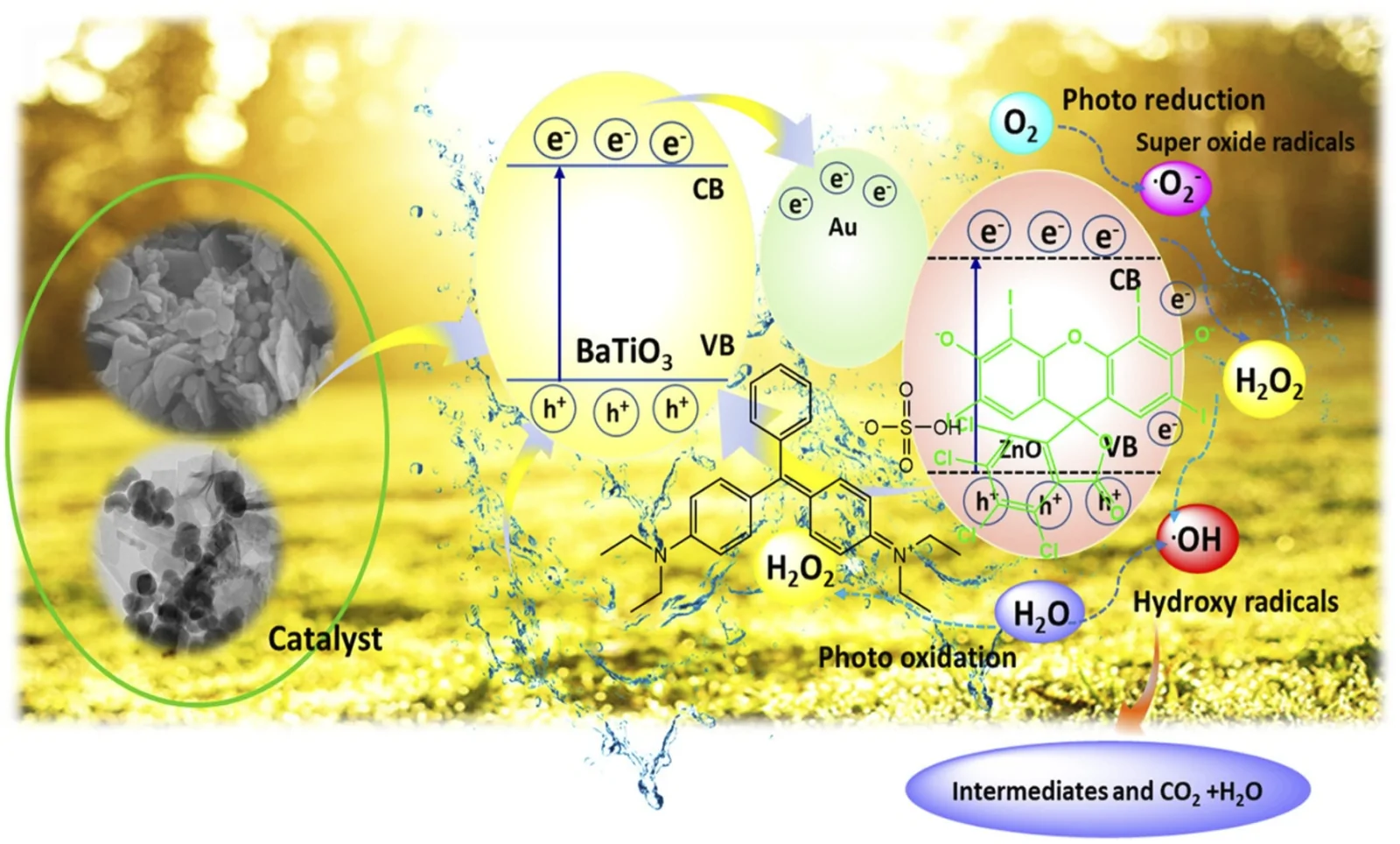
Dual heterojunction mechanism for photocatalytic degradation using arabic gum@BaTiO_3_–Au–ZnO. Reproduced with permission from ref. 35. Copyright, Elsevier B.V., 2025.

Nevertheless, one critical standpoint is that these proposed mechanisms should not be inferred from degradation efficiency or band-gap measurements alone. Evidence from photoluminescence quenching, transient photocurrent response, electrochemical impedance, other charge-transfer analyses, *etc.*, is required to establish whether improved charge separation is actually responsible for the observed enhancement. The relationship between gum incorporation, particle size, and optical properties also warrants careful interpretation. Although stabilisation of nanoparticles by a gum matrix can modify particle growth and consequently affect their optical and electronic behaviour, a lower reported band gap should not automatically be attributed to quantum confinement.^57^ This is because quantum-confinement effects depend on the particle dimensions relative to the characteristic exciton Bohr radius, whereas changes in band-gap behaviour in gum-containing nanocomposites may also originate from particle-size variation, structural defects, surface states, interfacial bonding, or electronic interactions between the organic matrix and the semiconductor. Thus, the molecular role of the gum should be considered as part of a broader structure–surface–property relationship rather than as an isolated source of photocatalytic enhancement.

One of the important developments in NGBNs is their application under direct sunlight, which provides a more practically relevant irradiation source than laboratory-scale artificial lamps and offers the potential to reduce the energy requirements of photocatalytic treatment. For example, in a case study, guar gum@CMC@SA@ZnO (at an optimized dose of 30 mg) was employed for the photocatalytic degradation of reactive yellow, red, and blue under natural sunlight exposure. In 240 minutes, over 94% of these dye pollutants were successfully degraded,^58^ and this is consistent with what was achieved (96.85%) for the photocatalytic degradation of MB under natural sunlight using spherical kondagogu gum@TiO_2_, but in a lesser time (90 minutes).^59^ These results suggest that gum-based photocatalysts can be highly effective under sunlight. However, from a critical position, the reported percentages and reaction times should not be interpreted as direct evidence that one gum-nanoparticle system is intrinsically superior to another because the studies involved different dyes, catalyst compositions, dosages, and experimental conditions. A more meaningful comparison therefore requires consideration of the catalyst-to-pollutant ratio, sunlight intensity and spectral distribution, reaction volume, and kinetic parameters rather than degradation percentage alone.

The dependence of degradation on the catalyst dosage further illustrates the interplay between surface availability and light transport. Increasing the NGBN dose can initially increase the number of accessible active sites and the available surface area for dye adsorption, thereby increasing the concentration of pollutant molecules near photocatalytically active interfaces and potentially enhancing reactive oxygen species generation. This trend was observed for kondagogu gum@TiO_2_, where increasing the catalyst dose improved MB degradation until an optimum was reached.^59^ Beyond this point, however, excessive catalyst loading increased solution turbidity and hindered the penetration and effective utilisation of incident sunlight, resulting in a reduction or plateau in degradation efficiency. Thus, catalyst dosage should not be viewed simply as a parameter that increases the number of active sites; rather, an optimum exists between the availability of catalytic surfaces and the optical accessibility of those surfaces. This consideration is particularly important for translating laboratory-scale NGBN systems to larger treatment volumes, where light attenuation and reactor geometry may become increasingly important.

Recent studies have also suggested that the gum matrix may influence the photocatalyst performance beyond surface area and adsorption by affecting its structural and electronic properties. This was observed in carrageenan-locust bean gum@Ag-TiO_2_ (ref. 60) and guar gum-agar agar@N-doped ZnO^33^ photocatalytic activity, with improved dye degradation as shown in Table 1. Furthermore, from a molecular-level point of view, it was revealed that the TiO_2_ surface provides additional adsorption sites through surface OH groups and pores, while Ag NPs enhance surface activity and create localized electron-rich regions that promote π–π and donor–acceptor connections between the aromatic MB rings and the surface of NGBN. Additionally, the porous hydrogel-like network formed by the carrageenan-locust bean gum and guar gum-agar agar enables diffusion and entrapment of MB dye molecules within its framework.^33,60^ The findings are consistent with the potential of gum incorporation to modify the microstructure, particle growth, surface chemistry, and interfacial electronic environment of semiconductor nanoparticles. O-containing groups such as –OH, –COOH, and ether groups present in natural gums interact with nanoparticle surfaces with exposed metal centres, and this enhances dye adsorption by the NGBN to facilitate effective radical attacks.^33,60^ In addition, it is imperative to point out that the association between higher crystallinity and improved degradation does not necessarily imply a universal causal relationship, since crystallinity may simultaneously influence defect density, particle size, surface area, and charge-carrier dynamics.

The differences in reaction time among sunlight-driven systems further demonstrate why degradation efficiency should be considered together with the experimental context. Guar gum@Fe_3_O_4_ achieved more than 97% degradation within 120 min,^61^ compared with more than 90% reported for guar gum/Al_2_O_3_ and guggul gum@BiFeO_3_ after approximately 220 and 760 min, respectively.^62,63^ Although these results indicate strong sunlight-driven activity across different gum-based systems, the longer treatment time required by some materials indicates that high final removal does not necessarily correspond to faster photocatalytic kinetics. Differences in dye structure and concentration, catalyst loading, sunlight intensity, reactor configuration, adsorption contributions, and other experimental conditions summarized in Table 1 could all influence the observed outcome. Notably, the absence of appreciable MB degradation in the absence of sunlight for kondagogu gum@TiO_2_ provides evidence for the importance of photoactivation in that system,^59^ but such controls need to be consistently applied across studies to thoroughly distinguish genuine photocatalytic degradation from adsorption or other removal processes. In a nutshell, the sunlight-based studies demonstrate the practical promise of NGBNs, but their comparison highlights the need to optimise the catalyst-to-dye ratio, maintain adequate light penetration, and systematically distinguish adsorption from photocatalytic oxidation while reporting irradiation and kinetic parameters sufficiently to enable meaningful cross-study evaluation.

Moreover, while sunlight-driven NGBN photocatalysis offers an attractive alternative to artificial irradiation because of the widespread availability and renewable nature of solar energy, one critical limitation is that its practical applicability cannot be generalized without considering geographical and temporal variability. This is because solar irradiance and spectral distribution vary with location, latitude, season, time of day, cloud cover, atmospheric conditions, and local climate, which can substantially influence the photon flux available for photocatalytic reactions. Consequently, the photocatalytic performance of NGBNs demonstrated under sunlight at a particular location and period may not be directly transferable to other geographical regions or operating seasons, and this may complicate reliable scale-up/commercialization. Therefore, future studies should take the geographical location, irradiation conditions, solar irradiance, and exposure period into consideration and, where possible, the spectral characteristics of the incident sunlight to facilitate the comparison and assessment of geographical applicability.

Recent advances have also been seen in assisted photocatalytic degradation processes using H_2_O_2_, persulfate, *etc.* This is particularly relevant because H_2_O_2_ can participate directly in the generation of reactive oxygen species while also accepting photogenerated electrons, thereby potentially reducing electron–hole recombination. For xanthan gum@TiO_2_, for example, MO degradation increased from approximately 74% to 89% within 120 min following the addition of H_2_O_2_.^64^ A similar, although less pronounced, improvement was reported for methyl violet degradation using rod-like guar gum-crosslinked soya lecithin@ZVI under natural sunlight, where the degradation efficiency increased from approximately 70% to 81% in the presence of 0.135 mM H_2_O_2_.^37^ The broadly consistent improvement across these systems suggests that H_2_O_2_ can complement the surface and electronic effects introduced by the natural gum matrix. Photogenerated electrons can reduce H_2_O_2_ and promote the formation of highly reactive species such as ˙OH, while the additional electron-accepting pathway can compete with e^−^/h^+^ recombination. However, the extent of this contribution is expected to depend on the H_2_O_2_ concentration, catalyst surface chemistry, irradiation conditions, and the availability of photogenerated charge carriers; therefore, the enhancement cannot be assumed to arise solely from a general H_2_O_2_ effect across all NGBNs.

The role of H_2_O_2_ is further illustrated by the solar-driven degradation of reactive red and reactive turquoise using xanthan gum@TiO_2_, for which the addition of 400 mg L^−1^ H_2_O_2_ resulted in more than 90% degradation within 120 min, with reported pseudo-first-order rate constants of 0.0183 and 0.0151 min^−1^, respectively.^65^ These kinetic values provide stronger evidence of enhanced treatment performance than final degradation percentages alone because they account for the rate of pollutant removal during the reaction period. Nevertheless, the comparison should be made with caution because the reported systems involved different dyes and H_2_O_2_ concentrations, and therefore the absolute degradation efficiencies and rate constants cannot be interpreted as direct measures of the intrinsic superiority of one gum-based photocatalyst over another. More importantly, the available evidence suggests that the enhanced performance results from a combination of factors rather than from a single property of the gum.^64,65^ This is due to the fact that, in this context, H_2_O_2_ can provide an additional pathway for converting photogenerated electrons into reactive oxygen species. Thus, the observed enhancement is more appropriately viewed as a synergistic interaction between gum-mediated surface modification, semiconductor photoactivation, and H_2_O_2_-assisted reactive-species generation. The superior performance of H_2_O_2_-containing systems should not be attributed entirely to the natural gum, since H_2_O_2_ is itself a chemically active component that provides an additional route for reactive oxygen species generation and charge-carrier utilization, especially when viewed critically with a molecular lens. However, the available studies have not yet reported the quantitative separation of the relative contributions of enhanced adsorption, altered semiconductor properties, and H_2_O_2_-mediated oxidation. Therefore, the higher degradation efficiencies reported for H_2_O_2_-assisted NGBNs should be interpreted as the outcome of a coupled treatment mechanism rather than as evidence of an intrinsic improvement attributable to the gum alone. A key potential limitation from available studies lies in the fact that reliance on continuous H_2_O_2_ supply during photocatalytic degradation operation may increase the operational costs and limit practical large-scale wastewater treatment applications despite the enhanced dye degradation observed. Moreover, critical practical questions that need to be answered by future researchers include the fate of residual H_2_O_2_ in treated effluent, the environmental and economic implications of continuous peroxide dosing at process scale, and the potential formation of peroxide-derived secondary oxidants in complex wastewater matrices, which are not addressed in current studies.

Overall, looking at the general performance trends across collected studies in Table 1, natural gum-based nanomaterials demonstrated consistently high degradation efficiencies. Specifically, there is a consistent trend of high photocatalytic degradation efficiency among the 5 most commonly used natural gums, with arabic gum topping the chart, followed by guar gum, xanthan gum, karaya gum, and tragacanth gum. However, the optimal NGBN dose exhibited substantial variability, ranging from 0.5 mg (ref. 66) to 1000 mg,^51^ with several formulations achieving high degradation efficiencies at relatively low loadings (<200 mg). Nonetheless, these values should not be interpreted directly as evidence that some NGBNs are intrinsically more efficient than others because the reported optimum dose is highly dependent on the experimental conditions summarized in Table 1. In particular, the absolute catalyst mass cannot be meaningfully compared without considering the reaction volume, initial dye concentration, catalyst-to-pollutant ratio, specific surface area, particle size, surface chemistry, and reactor configuration. A 20 mg catalyst dose, for example, may represent a substantially different catalytic loading depending on whether it is applied to a few millilitres or hundreds of millilitres of dye solution. Similarly, increasing the NGBN loading can initially increase the number of accessible active sites and enhance pollutant adsorption, but excessive loading may promote particle aggregation, increase solution turbidity, and enhance light scattering, thereby restricting photon penetration and reducing the effective utilisation of the catalyst. The optimum dose is therefore better understood as a balance between the availability of catalytic/adsorptive sites and the optical accessibility of those sites rather than as a universal property of the NGBN.

A similarly wide variation was observed in treatment time, ranging from ultra-fast degradation within 1 min (ref. 66) to 760 min.^63^ Although such differences demonstrate the potential of NGBNs to operate over a broad range of reaction times, they also highlight the difficulty of ranking photocatalysts solely according to their reported degradation percentages. The reaction time is influenced by irradiation intensity and wavelength, initial pollutant concentration, dye structure and concentration, catalyst loading, pH, reactor geometry, adsorption behaviour, and the morphology and electronic properties of the NGBN. For solar-driven systems, additional variability may arise from changes in solar irradiance and spectral distribution during the experiment. Thus, a material achieving a high degradation percentage within a short time under intense irradiation cannot automatically be considered superior to a material requiring a longer treatment period under natural sunlight or substantially different pollutant and catalyst concentrations. Comparison of kinetic parameters, together with catalyst and pollutant loading and irradiation conditions, would therefore provide a more reliable assessment of intrinsic photocatalytic performance.

The wide divergence in catalyst dose and treatment time consequently reflects not only differences among NGBN formulations but also substantial differences in experimental design and optimisation conditions across the literature summarized in Table 1. This represents an important standardisation challenge because the absence of consistently reported catalyst concentrations, reaction volumes, pollutant concentrations, irradiation intensity, spectral characteristics, pH, dark adsorption periods, and kinetic parameters limits direct cross-study comparison. Thus, the observation that many NGBNs achieve high degradation efficiencies under different conditions should be interpreted as evidence of promising performance across diverse experimental systems rather than as definitive proof of universal robustness or broad applicability. In particular, high removal efficiencies may result from different combinations of adsorption, light absorption, reactive oxygen species generation, and charge-carrier dynamics, whose relative contributions are not always separated experimentally.

The relationship between morphology and degradation performance presents a similar interpretative challenge. Although the available studies do not indicate a simple relationship between morphological shape or IUPAC pore classification and degradation efficiency, the recurrent observation of spherical and mesoporous architectures is noteworthy. Such structures could favour pollutant diffusion, increase the accessibility of internal and external active sites, and facilitate interaction between the catalyst and incident radiation. Nevertheless, these effects should be regarded as plausible structure–performance relationships rather than universal explanations, because morphology, pore characteristics, particle size, surface area, crystallinity, and electronic properties often vary simultaneously. Isolating the specific contribution of spherical morphology or mesoporosity would therefore require comparative studies in which other material and operational variables are controlled.

Additionally, despite the recent advances reported in the foregoing paragraphs, the degradation efficiency alone is not an adequate metric for comparing photocatalysts like NGBNs because it depends heavily on experimental parameters. Thus, more appropriate metrics, such as quantum efficiency, mineralization efficiency, and TOC removal, are needed to complement the degradation efficiency in future studies.

## 6. Photostability and reusability potential

The possibility of reusing spent photocatalysts speaks volumes about their photostability, potential industrial applicability, economic value, and eco-friendliness, as uncontrolled disposal of spent photocatalysts can collaterally contaminate the environment and defeat the original intention of a win–win remediation strategy. Moreover, recyclability evaluation supports the circular economy initiative.^91–93^ In the subsequent paragraphs, case studies on the reusability and photostability of various NGBNs for the photocatalytic degradation of different dye pollutants are analyzed and presented in Table 2 alongside various eluting/regenerating agents employed, the number of cycles, and the degradation efficiency in the last cycle.

**Table 2:** Overview of NGBN recyclability performance^a^

Natural gum-based nanomaterial	Dye pollutant degraded	Eluting/regenerating agent	% degradation in the 1^st^ cycle	No. of cycles (n)	% degradation after last cycle	Ref.
Arabic gum@TiO_2_	Methylene blue	NA	98.85	3	98.12	51
Karaya gum@TiO_2_	Methylene blue	NA	>85	3	77	52
Arabic gum@TiO_2_	Methylene blue	NA	>90	3	80	52
Guar gum@MgB_2_@Ru	Crystal violet	Distilled H_2_O	99.2	5	>85	55
Almond gum@BiO	Congo red	NA	90.21	4	87	38
Gum arabic@CuFe_2_O_4_–Ag	Methylene blue	NA	90	3	71	70
Gum acacia@TiO_2_	Rhodamine-B	EtOH and Milli-Q H_2_O	100	5	77	71
Gum acacia@TiO_2_	Methyl orange	EtOH and Milli-Q H_2_O	92	5	71	71
Guar gum@Fe_3_O_4_	Crystal violet	EtOH and distilled H_2_O	98.94	6	>80	61
Guar gum-crosslinked soya lecithin@ZVI	Methyl violet	NA	81	6	69	37
Tragacanth gum@TiO_2_	Crystal violet	Distilled H_2_O	89.2	5	88.12	73
Agar gum@Ag@cellulose	Malachite green	NA	98.35	5	87.54	74
Fenugreek gum@DDM@TiO_2_	Congo red	Distilled H_2_O	95.27	4	90.5	75
Karaya gum@g-C_3_N_4_@ZnO@SiO_2_	Trypan blue	Double-distilled H_2_O	92.22	4	80.03	76
Guggul gum@BiFeO_3_	Magenta-O	0.1 N HCl	96.65	5	76.2	63
Guar gum-agar agar@N-doped ZnO	Yellow dye	Distilled H_2_O and acetone	92	6	88.5	33
Guar gum@carboxymethyl cellulose@ZnO–Fe_2_O_3_	Crystal violet	Distilled H_2_O and acetone	92	6	>80	
Guar gum@carboxymethyl cellulose@ZnO–Fe_2_O_3_	Methylene blue	Distilled H_2_O and acetone	95	6	>90	31
Gellan gum@TiO_2_	Methylene blue	NA	100	3	78	82
Xanthan gum@agar@ZnO	Malachite green	NA	99.45	5	90.92	57
Xanthan gum@Cu–Fe_3_O_4_	Methylene blue	Distilled H_2_O and EtOH	98	7	90	66
Locust bean gum@Zr(iv) selenophosphate	Fast sulphon black	NA	90	5	>80	85
Gum acacia-crosslinked-poly(acrylamide) hydrogel@C_3_N_4_/BiOI	Crystal violet	Distilled H_2_O and EtOH	88	5	68	53
Tragacanth gum@CeO_2_@rGO	Methylene blue	Distilled H_2_O	∼91	4	>80	89
Poly(vinyl alcohol)-tragacanth gum@TiO_2_	Methylene blue	Distilled H_2_O	95.54	5	79.59	50

a NA = not available/reported.

For instance, in a case study,^51^ the photostability and recyclability potential of arabic gum@TiO_2_ for the photocatalytic degradation of methylene blue were evaluated over 3 cycles of experiment. It was noted that after the three consecutive cycles, the photocatalytic degradation efficiency of the NGBN remained 98.12%, which is just an infinitesimal reduction of 0.73% when compared to the 98.85% recorded in the first cycle. It was opined that the slight reduction in removal might be a result of intermediates absorbed on the surface of NGBN, as well as agglomerations that may form from particles and benefit the recombination and/or separation of charge carriers in the reaction medium by reducing the effective SA and numerical strength of active sites that are responsible for photon, electron, and radical generation. Moreover, the study outcome shows good photostability, as no alterations in the crystalline structure were noticed following the three rounds of photocatalytic degradation operation. Specifically, arabic gum@TiO_2_ diffractograms prior to and following the photocatalytic reuse test showed a very comparable profile, as shown in Fig. 9a, demonstrating that the NGBN's crystalline structure was unaffected. This supports arabic gum@TiO_2_'s exceptional capacity to degrade methylene blue in the three successive rounds of reuse.^51^ Nonetheless, one critical reservation is that the claim of eco-friendliness and exceptional stability cannot be wholly believed, as further study on possible mass loss and leaching of metals like Ti and Fe is needed to corroborate such a conclusion. Similar remarkable photostability and recyclability were also recorded for karaya gum@TiO_2_ and arabic gum@TiO_2_ for the photocatalytic degradation of methylene blue. From the SEM and XRD analysis of the spent NGBN, it was observed that the spherical and crystalline architectural morphology of the two natural gum-based nanomaterials remained intact after three recyclability studies. Notably, it is obvious that the interaction between the gums and TiO_2_ that is formed enables/preserves the structural integrity of NGBNs and thwarts morphological changes that can make reusing difficult, and this reinforces the physicochemical stability idea of natural gum-based nanomaterials for improved wastewater treatment in real-world applications.^52^ In a comparable recyclability study, just a minor reduction in the Congo red degradation efficiency (from 90.21% to 87%) was seen using almond gum@BiO after four successive reusability rounds. The slight drop in the degradation efficiency was due to minor NGBN loss during each separation procedure. Amazingly, the XRD analysis confirmed that the recycled material's crystalline structure remains unchanged, as established by the persistence of the peaks following reuse, and this signifies the remarkable durability of almond gum@BiO.^38^ Meenu *et al.* also noted that there was no structural damage in spent gum arabic@CuFe_2_O_4_–Ag after three consecutive recyclability tests for MB degradation operations,^70^ and this confirms the photostability of NGBMs. Nevertheless, according to research findings, it has been opined that the buildup of small molecules on the NGBM surface, which may impede the accessibility of light, as well as reduced porosity of the material, is part of the logical scientific reasons for the minor decrease in degradation output over successive cycles. Additionally, while structural stability is inherently beneficial for multiple recoverability and recyclability efficiency, excessively strong interfacial interactions may reduce the accessibility of catalytic sites or limit mass transfer within the porous structure. This could ultimately compromise the catalytic efficiency at an industrial reusability level despite the apparent structural stability and recyclability efficiency on a lab scale.

**Fig. 9:**
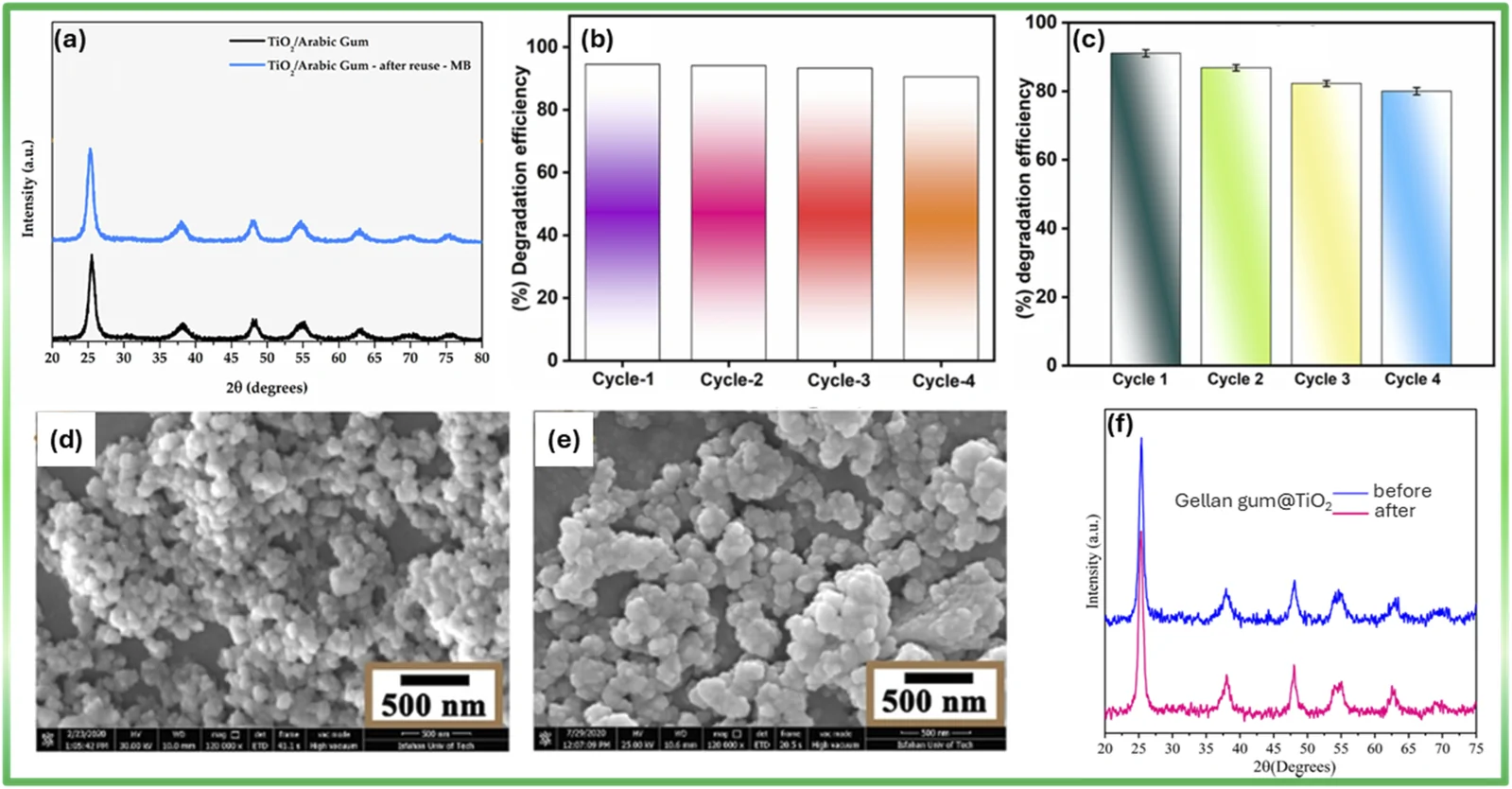
(a) XRD patterns of arabic gum@TiO_2_ after photostability and recyclability test. Reproduced from ref. 51 which is open access and permits unrestricted use of materials under the terms of Creative Commons Attribution (CC-BY). (b) Recyclability efficiency of fenugreek gum@DDM@TiO_2_ for Congo red degradation over four rounds. Reproduced with permission from ref. 75. Copyright, The Korean Society of Industrial and Engineering Chemistry. Published by Elsevier B.V., 2024. (c) Recyclability efficiency of karaya gum@g-C_3_N_4_@ZnO@SiO_2_ for trypan blue degradation over four rounds. Reproduced with permission from ref. 76. Copyright, Elsevier B.V., 2025. Stability analysis of tragacanth gum@TiO_2_ on crystal violet: FE-SEM images (d) before use and (e) after five cycles of reuse. Reproduced with permission from ref. 73. Copyright, Elsevier B.V., 2021. (f) XRD patterns of gellan gum@TiO_2_ after photostability and recyclability tests for methylene blue degradation. Reproduced from ref. 82 which is open access and permits unrestricted use of materials under the terms of Creative Commons Attribution (CC-BY).

In contrast, Kumari *et al.* differ in their own observation, where the reusability potential of gum acacia@TiO_2_ was tested after washing with EtOH and Milli-Q H_2_O following each round of photocatalytic degradation operation. Specifically, it was noted that despite the fact that the regenerated spent NGBM gave over 70% efficiency for both MO and RhB after five cycles, some kind of structural changes were visible. Evidently, over consecutive reusability, the porosity of gum acacia@TiO_2_ diminished, which was attributed to the presence of residual dye that is not totally desorbed even after washing during the regeneration process. This then results in the partial obstruction of some active sites, less dye diffusion, and reduced light penetration to the gum acacia@TiO_2_ site.^71^ This means that gum acacia@TiO_2_ is strongly reusable, but not completely photostable, and this raises a concern about its potential reusability in real-world effluent treatment or harsh conditions. Moreover, from our own perspective, given that other studies have recorded good structural stability and photostability, the slightly contrary outcome here might be due to the imbalance ratio between gum acacia and TiO_2_ during the synthesis. Additionally, while the reusability performance suggests the economic potential of the material, the lack of a quantitative study on the NGBN catalyst mass loss after multiple reusability cycles undermines the pilot-scale potential, and this is a critical limitation for commercialization acceptance.

In another research, Mallakpour *et al.* explored the recyclability and photostability potential of tragacanth gum@TiO_2_ for the photocatalytic degradation of crystal violet. Notably, after five successive rounds of reusability study, the spent NGBN that was just washed with distilled H_2_O still delivered 88.12% dye degradation efficiency with no serious structural damage, as seen in Fig. 9d and e, where the SEM image of the reused spent material is very similar to the pristine one. This confirms the photostability of tragacanth gum@TiO_2_.^73^ This is similar to what was observed after fenugreek gum@DDM@TiO_2_ (ref. 75) and karaya gum@g-C_3_N_4_@ZnO@SiO_2_ (ref. 76) delivered >80% Congo red and trypan blue degradation efficiency after 4 cycles of reusability study, as shown in Fig. 9b and c, without severe structural compromise.^75^ Moreover, no morphological or structural changes were noticed after guar gum@Fe_3_O_4_ was reused 6 times for the photocatalytic degradation of crystal violet, affirming the NGBN photostability and potential for real dye effluent treatment.^61^ From our own perspective, the strong interactions and hydrogen bonding between the gums and nanoparticle components, while beneficial for structural stability and recyclability, may also introduce inherent limitations. The enhanced structural rigidity can hinder efficient regeneration, as strongly adsorbed intermediates or degradation by-products may not be easily desorbed, leading to gradual active site blockage over repeated cycles. This could ultimately compromise catalytic efficiency despite apparent structural stability. Furthermore, such tightly bound architecture may be more susceptible to irreversible deactivation pathways, including pore blocking and reduced surface reactivity, which are not readily reversed through conventional regeneration methods. From a process perspective, the inability of these materials to lose form, as confirmed by various XRD, SEM, and FTIR analyses, may also limit their adaptability in shaping, post-synthetic modification, or integration into scalable systems where some degree of structural flexibility is often required.

Overall, the data presented in Table 2 revealed that natural gum-based nanomaterials exhibit consistently good reusability and operational stability trends. The finding shows that the NGBN can be reused for an average of ∼5 consecutive cycles (and up to 7) while retaining approximately 88% of its original activity, with a modest average efficiency loss of only ∼12%. Additionally, more than 60% of the reported systems still achieve ≥85% degradation efficiency in the final cycle, and about 90% remain above the practical threshold of 80% efficiency. Furthermore, while not many studies reported the eluting/regenerating agent used, a consistent trend of using distilled water and ethanol was observed. This is highly eco-friendly and economical. These results demonstrate that natural gums not only enhance the photocatalytic degradation performance of the NGBN but also impart remarkable cyclic and photo-stability to the nanocomposites, making them highly promising for cost-effective and sustainable photocatalytic wastewater treatment applications.

However, from our critical perspective, several important research gaps and limitations emerge from the current body of work. The first critical limitation noticed is the lack of standardized protocols for evaluating catalyst recyclability, which significantly undermines cross-study comparability. Regeneration strategies vary widely, ranging from centrifugation and filtration to ethanol washing and water rinsing, yet the underlying regeneration mechanisms are rarely investigated. Moreover, a consistent decline in performance is observed upon reuse and, in some cases, drops below 80%. From our own perspective, this clearly indicates that progressive NGBN catalyst deactivation rather than just mere efficiency reduction, as concluded in many studies. Potential causes of deactivation, such as photocorrosion of metallic nanoparticles, pore blockage by dye intermediates, and metal leaching that can occur during multiple reuses, remain underexplored. Specifically, photocorrosion is an important potential pathway for semiconductor (especially II–VI materials like CdS in NGBNs) photocatalyst deactivation under prolonged irradiation. It occurs when photogenerated charge carriers participate in undesirable redox reactions involving the semiconductor itself, resulting in structural alteration, dissolution of the active phase, and loss of photocatalytic activity and reusability.^94–96^ Although direct studies of photocorrosion in NGBN remain limited, ZnO and CdS provide representative examples of how such deactivation may occur. For ZnO in NGBN, prolonged irradiation may promote photocorrosion and dissolution of the ZnO phase, particularly when charge-carrier recombination limits the effective transfer of photogenerated electrons and holes to surface reaction sites, potentially resulting in Zn-ion leaching and progressive loss of active material.^95^ Similarly, as shown in Fig. 10, in the NGBN photocatalytic system containing CdS, accumulated photogenerated holes can oxidize surface S^2−^ species, leading to sulfur oxidation and photo-dissolution of CdS; dissolved O_2_ may further contribute to this process.^94,97^ In NGBNs, such semiconductor degradation could be accompanied by weakening or swelling of the gum matrix, facilitating nanoparticle/metal-ion leaching and blocking or alteration of active sites by reaction intermediates. Consequently, photocorrosion, leaching, and surface fouling may collectively contribute to progressive catalyst deactivation and the reduced photocatalytic efficiency often observed during repeated use.^94–96^ These mechanisms, however, require direct experimental verification for ZnO/gum, CdS/gum, and other NGBN systems. This is a crucial research gap that needs to be filled in future work. Moreover, there is a notable absence of quantitative stability metrics, as most studies report only percentage degradation without accounting for catalyst mass loss or metal ion release during recovery or recycling. This study gap is non-trivial as it raises concerns regarding the risk of secondary environmental contamination and restricts the environmental relevance of current findings.

**Fig. 10:**
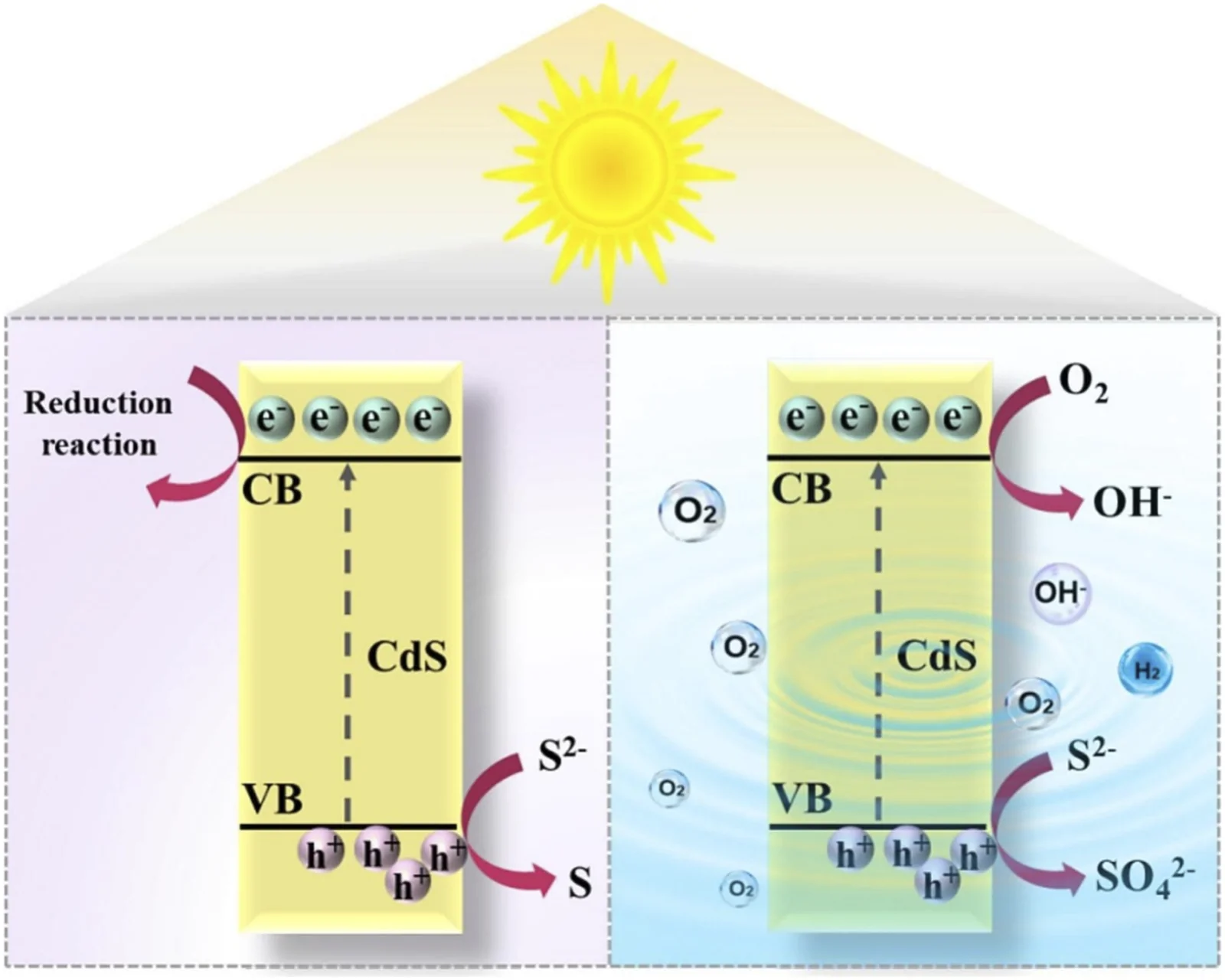
Schematic of the typical photocorrosion mechanism. Reproduced with permission from ref. 94. Copyright, Elsevier B.V., 2025.

Another important issue observed is the lack of kinetic studies throughout the recyclability experiments. Although percentage degradation efficiencies are routinely presented, the evolution of reaction kinetics across cycles, such as changes in rate constant or reactive species generation, is rarely monitored, limiting a deeper understanding of NGBN catalyst stability.

Moreover, the limited inclusion of structurally complex pollutants, such as mixed dye contaminant systems or real wastewater systems, highlights a disconnect between laboratory evaluations and practical application scenarios within the context of recoverability, regenerability, stability, and reusability. Given the presence of competing ions, natural organic matter, and fluctuating physicochemical conditions in real wastewater systems, the reported performances under simplified conditions observed across studies may be overly optimistic. Furthermore, most regeneration and recovery approaches are inherently laboratory-scale, with heavy dependence on centrifugation and similar batch techniques that are impractical for industrial deployment. The lack of emphasis on scalable strategies such as continuous-flow operation, catalyst immobilization, or magnetic separation further constrains industrial applicability potential. All of these identified research gaps need to be addressed by future researchers to advance the NGBNs' photocatalytic technique and compete with existing wastewater remediation methods.

## 7. Toxicological analysis and ecological structure–activity relationships

For a holistic evaluation of the effectiveness of any material, degradation system, and its environmental consequences from an ecological and industrial perspective, a toxicological study is very essential.^35^ The toxicological study is crucial, as the intermediate compounds formed during the photocatalytic degradation process of the dye may be more toxic than the dye pollutant itself.^52^ Moreover, this study not only validates the biocompatibility of the material but also gives quality information about the potential toxicity of the material (natural gum-based nanomaterial in this case) using Eco-QSAR (Ecological Quantitative Structure–Activity Relationship) platforms like the Ecological Structure–Activity Relationships (ECOSAR) software.

For example, in a particular study, after the photocatalytic degradation of rose bengal using arabic gum@BaTiO_3_–Au–ZnO, the potential toxicity of the degradation byproducts on fish, daphnids, and green algae was examined using the ECOSAR predictive software. Then, the toxicity was categorized following the Globally Harmonized System of Classification and Labelling of Chemicals (GHS), utilizing acute toxicity metrics such as LC_50_, EC_50_, and chronic toxicity values (ChV). Notably, the toxicity assessments of the degradation product with *m*/*z* 112 showed that it poses no ecological risk. Both acute and chronic tests classified it as non-toxic to fish, daphnia, and green algae, with LC_50_ values of 11 500 mg L^−1^ (fish, 96 h) and 5740 mg L^−1^ (daphnia, 48 h), and an EC_50_ of 2480 mg L^−1^ (green algae, 96 h). The corresponding chronic values were 965 mg L^−1^ for fish, 388 mg L^−1^ for daphnia, and 485 mg L^−1^ for green algae. In contrast, the *m*/*z* 122 product showed harmful acute toxicity toward all three aquatic organisms, indicating a moderate environmental impact. Similarly, the *m*/*z* 158 product exhibited harmful effects on daphnia and green algae, suggesting potential ecological concern. Overall, these findings highlight clear differences in toxicity among degradation products. Importantly, this study underscores the need to monitor RB and its transformation products to mitigate ecological risks.^35^ In a similar study, where karaya gum@g-C_3_N_4_@ZnO@SiO_2_ was employed for the photocatalytic degradation of trypan blue and Congo red dye pollutants, Eco-QSAR analysis was performed using the established computational software (ECOSAR) to evaluate the potential toxicity of the intermediate degradation products. Specifically in this study, the ecotoxicity was quantified using LC_50_ and EC_50_ metrics. The results show that Congo red is highly toxic to aquatic organisms, with acute LC_50_ values of 5.41 mg L^−1^ for daphnia, 5.69 mg L^−1^ for fish, and an EC_50_ of 13.7 mg L^−1^ for algae. Chronic toxicity was even more pronounced, with chronic values of 0.169 mg L^−1^ (daphnia), 0.015 mg L^−1^ (fish), and 1.10 mg L^−1^ (algae). The Congo red degradation product (*m*/*z* 258) exhibited severe acute and chronic toxicity, particularly toward fish and daphnia. Increased toxicity was linked to harmful functional groups formed after the ring structure by radicals generated by karaya gum@g-C_3_N_4_@ZnO@SiO_2_ during the photocatalytic degradation operation. In contrast, several degradation products, Congo red (*m*/*z* 118, 73, 60) and trypan blue (*m*/*z* 104, 60), were less toxic, indicating reduced environmental risk. Although fish showed fewer chronic effects, daphnia remained the most sensitive species. These findings underscore the need for a broad assessment of the ecological risk of NGBNs and dye degradation intermediates by future researchers.^76^

Furthermore, one critical limitation is that while the current studies employed ECOSAR for predictive ecotoxicity assessment, quantitative model-validation metrics or prediction accuracy were not reported for the ECOSAR predictions. Therefore, the reported LC_50_, EC_50_, and chronic toxicity metric values should be interpreted as predicted estimates rather than experimentally validated toxicity metric values. This limitation highlights the need for future studies to complement Eco-QSAR predictions with experimental ecotoxicity assays and to report appropriate validation and applicability-domain information.

In another case study, the cytotoxicity of gum arabic/guar gum@CuO employed for the photocatalytic degradation of methylene blue and crystal violet was evaluated by the MTT [3-(4,5-dimethylthiazol-2-yl)- 2,5-diphenyl tetrazolium bromide] assay using the Caco-2 cell culture model. Notably, with varied concentrations, the NGBN displayed cell viability around 93.73–99.97 %. Moreover, at a concentration of 0–2 mg mL^−1^, the NGBN showed an insignificant difference in cell viability. The gum arabic/guar gum@CuO demonstrated low toxicity in contrast to ordinary nanoparticles without the natural gum. It was opined that this may be because the natural gum polymeric 3D network prevented the nanoparticles' ionic structures from degrading,^32,33^ and this shows the positive impact of natural gum in NGBNs. Similarly, Sharma *et al.* assessed the cytotoxicity of gaur gum@CuO used for the degradation of malachite green on three cell lines (CHO-K1, KB, and C6) by using different concentrations after 24, 48, and 72 hours of exposure. Based on the findings, the authors concluded that NGBN has no cytotoxic effect on CHO-K1 cells, rat glioma C6 cells, and oral cancer cells KB across a wide range of temperatures, concentrations, and exposure times.^86^

In another toxicological study, toxicity bioassays were carried out using *A. salina* microcrustacean, where microcrustaceans were grown in synthetic saline (38.0 g L^−1^) for 48 hours under regulated oxygen and illumination conditions. The death rate of microcrustaceans was assessed after 24 hours of contact with a methylene blue solution treated with karaya gum@TiO_2_ and arabic gum@TiO_2_ photocatalysts under light exposure. The result shows that the percentage of live microcrustaceans was >80% after 24 hours, as compared to the untreated dye solution. This outcome established that both the photocatalytic degradation byproducts and the NGBN photocatalyst employed do not display toxicity.^52^ In a comparable toxicological experiment on methylene blue solution from gellan gum@TiO_2_ photocatalytic degradation operation, toxicity bioassays were performed using *A. salina* microcrustaceans, where microcrustaceans were grown in synthetic saline (38.0 g L^−1^) for 48 hours under regulated oxygen and illumination conditions. The death rate of microcrustaceans was assessed after 24 hours of contact with a methylene blue solution that had been treated with the gellan gum@TiO_2_ photocatalyst under light exposure. The death rates were found to be negligible. There were no negative effects from the photocatalytic degradation solutions. Since the mortality of nauplii was nearly identical to that of the control sample, which maintained 100% of the brine shrimp alive for 24 and 48 hours, the authors suggest that the degradation intermediates have little or no toxicity.^82^ It also indicates that used gellan gum@TiO_2_ did not impart any toxicity to the treated solution, thus confirming its eco-friendliness.

In another case study,^38^ the dose-dependent cytotoxicity of almond gum@BiO (employed for the photocatalytic degradation of Brilliant green and Congo red dyes) toward NIH/3T3 cells was evaluated over 72 h. It was observed that cell viability decreased with the increasing almond gum@BiO concentrations, with an IC_50_ value of 32.12 µg mL^−1^. Lower doses supported higher cell survival, indicating reduced cytotoxicity at minimal exposure levels. Despite this, almond gum@BiO also induced notable morphological changes, attributed to its high SA-to-volume ratio. While these findings suggest potential cytotoxic effects, FDA/EtBr staining showed predominantly live cells, confirming potential biocompatibility. Moreover, extended incubation improved cellular activity, suggesting that almond gum@BiO may even support cell proliferation.^38^

Overall, the available evidence suggests that several NGBNs exhibit low cytotoxicity and favourable biocompatibility under the specific experimental conditions investigated. However, this evidence remains limited to a relatively small number of studies, specific cell lines, aquatic organisms, exposure concentrations, and exposure durations. Moreover, the observation of toxicity in some photocatalytic degradation intermediates demonstrates that effective dye degradation does not necessarily correspond to complete detoxification. Therefore, the environmental safety of NGBNs cannot still be generalized across different gum matrices, embedded metal/metal oxide nanoparticles, pollutants, and environmental conditions. In particular, chronic toxicity, endocrine-disrupting effects, long-term environmental persistence, bioaccumulation, and the fate and potential release of metal nanoparticles or metal ions from gum matrices remain insufficiently investigated. Future studies should therefore combine acute and chronic ecotoxicity assessments with mechanistic toxicity studies, degradation-product identification, long-term environmental fate analysis, and experimental validation of Eco-QSAR predictions. Such comprehensive assessments are necessary to establish whether the environmental benefits of NGBNs are sustained beyond their photocatalytic performance and short-term biocompatibility.

## 8. Technology readiness level

The technology readiness level (TRL) framework provides a standardized nine-level scale, from TRL 1 (basic scientific principles) to TRL 9 (fully demonstrated and commercially deployed technology), to evaluate a technology's maturity relative to its intended real-world application, as shown in Fig. 11.^98,99^ The framework provides a structured basis for assessing technological progression and reducing uncertainty in implementing complex systems.^88,89^ In the present review, the conventional TRL framework was adapted specifically to NGBN photocatalytic systems for wastewater treatment. The assessment considers the progressive development of the technology from fundamental scientific understanding and laboratory proof of concept to validation under relevant conditions, pilot-scale demonstration, and eventual industrial deployment. This approach is consistent with established TRL methodologies, in which advancement to a higher level requires demonstrable achievement of increasingly demanding technical and operational requirements rather than performance based solely on the laboratory degradation efficiency.^98–101^ Recent assessments of photocatalytic water-treatment technologies similarly indicate that most reported systems remain within the research and laboratory-validation stages, while progression toward pilot and industrial application is constrained by reactor engineering, realistic water matrices, long-term operation, and process feasibility.^102–106^

**Fig. 11:**
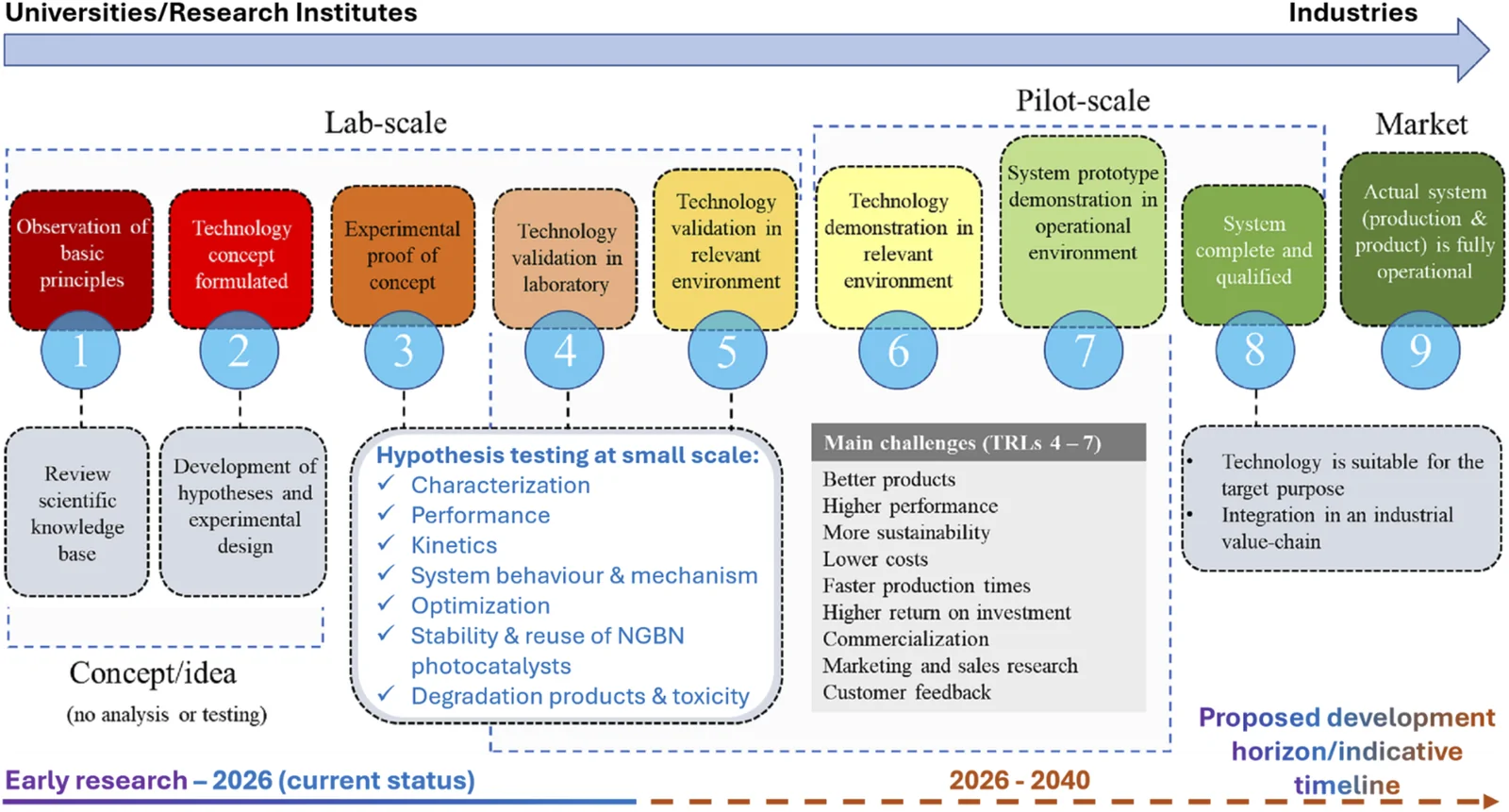
TRLs applied to dye treatment/photocatalytic technique using nanomaterials and proposed development horizon/indicative timeline for NGBN-based photocatalytic wastewater treatment from early research, current status, to potential commercial deployment. Adapted with permission from ref. 107. Copyright, Elsevier B.V., 2023.

For the present review, the criteria applicable to each TRL were adapted from related dye effluent treatment studies^93,107–111^ to reflect the specific development requirements of NGBN-based photocatalytic wastewater treatment. At TRL 3–4, where the majority of the reviewed NGBN systems are currently positioned, the applicable criteria include: (i) characterization of the NGBN photocatalyst, including its physicochemical, structural, morphological, and optical properties; (ii) evaluation of photocatalytic performance based on percentage degradation/removal, degradation kinetics, and, where available, mineralization; (iii) analysis of the fundamental behaviour of the photocatalytic system, including the effects of catalyst dosage, pollutant concentration, pH, irradiation time, and temperature, together with kinetic modelling and investigation of the photocatalytic mechanism; (iv) optimization of the NGBN photocatalytic system under controlled laboratory conditions; (v) evaluation of catalyst stability and recovery through repeated-use experiments; and (vi) preliminary assessment of degradation products and environmental safety through transformation-product identification and toxicity or ecotoxicity studies, where available.

Based on these criteria, NGBN-based photocatalytic systems for dye degradation are predominantly positioned at TRL 3–4, rather than broadly across TRL 1–4. TRL 1–2 represents the fundamental scientific and conceptual foundation of the technology, including understanding of photocatalytic ROS generation and the roles of natural gums in nanoparticle stabilization, dispersion, surface modification, and photocatalytic performance.^112^ Natural gums such as guar, xanthan, arabic, and karaya gums contain functional groups, including hydroxyl, carboxyl, and amino groups, that can participate in chelation, reduction, stabilization, and templating during nanocomposite formation. Various photocatalytic components, including TiO_2_, ZnO, SnO_2_, CuO, Fe_2_O_3_, graphene oxide (GO), and reduced graphene oxide (rGO), have been incorporated into gum matrices.^28,32,35,72,75,76,89^ These studies establish the scientific basis for the development of NGBN photocatalysts and therefore represent the foundation of TRL 1–2.

However, the majority of reported studies have progressed beyond the conceptual stage and have demonstrated measurable photocatalytic activity under laboratory conditions. Consequently, they satisfy the principal requirements of TRL 3–4. At TRL 3, proof-of-concept^98,113^ studies have demonstrated the degradation of model dyes, including MB,^90^ MO,^84^ malachite green,^50^ Congo red,^66^ and rhodamine B,^84^ using gum/TiO_2_,^51^ gum/ZnO,^58^ gum/Fe-based nanoparticles,^66^ and ternary nanocomposite systems.^53,89^ At TRL 4, these systems have generally undergone laboratory optimization involving catalyst dosage, initial dye concentration, pH, irradiation time, and reaction kinetics. Controlled UV or visible-light batch reactors remain the dominant experimental configuration. In addition, several studies have reported catalyst reuse, with photocatalytic activity generally retained at greater than 75% over approximately 3–7 cycles.^52,66^ These findings provide evidence of preliminary operational stability but remain insufficient to demonstrate extended process reliability.

Furthermore, some studies have incorporated additional oxidation processes to enhance photocatalytic degradation. For example, H_2_O_2_-assisted systems involving Fenton or photo-Fenton reactions have achieved near-complete dye removal over shorter treatment periods.^65^ Nevertheless, these experiments have predominantly been conducted using idealized model solutions under controlled laboratory conditions. Thus, the high degradation efficiencies reported in such studies should not be interpreted as evidence of advanced technological readiness. Rather, they demonstrate improved laboratory performance within the TRL 3–4 range.

Importantly, a limited number of investigations have moved beyond simple measurement of parent-dye removal by identifying degradation intermediates using techniques such as liquid chromatography-mass spectrometry (LC-MS) and gas chromatography-mass spectrometry (GC-MS), together with preliminary toxicity assessment. These studies represent an important progression toward TRL 4 because they begin to address whether photocatalytic treatment results in detoxification rather than merely disappearance of the parent pollutant. However, comprehensive toxicological assessment remains uncommon, particularly with respect to chronic toxicity, endocrine-disrupting potential, mutagenicity, persistence, bioaccumulation, and long-term environmental effects. Therefore, these studies provide valuable supporting evidence for laboratory validation but do not yet satisfy the broader environmental and operational requirements associated with higher TRLs.

Similarly, studies conducted under natural sunlight provide an important step toward more realistic operating conditions. Some NGBN systems have been evaluated using natural or unfiltered sunlight in open or semi-outdoor configurations, thereby reducing dependence on controlled laboratory light sources.^37,64^ Nevertheless, photon flux, irradiation intensity, cloud cover, season, geographical location, and exposure duration are inherently variable under natural sunlight. Moreover, most of these investigations have still used synthetic dye solutions at laboratory scale rather than real industrial or municipal wastewater. The absence of continuous-flow operation, wastewater fouling assessment, long-term catalyst stability, and large-scale reactor validation means that such studies should generally be regarded as advanced TRL 4 or, in limited cases, preliminary TRL 5, rather than as evidence of mature pilot-scale technology.

Environmental-risk assessment using Eco-QSAR and ECOSAR can further strengthen the environmental evaluation of NGBN photocatalytic systems. Such approaches have been used to predict the acute and chronic toxicity of dye degradation intermediates toward organisms such as fish, daphnids, and green algae. These assessments are valuable because they can identify potentially hazardous transformation products that may not be apparent from conventional dye-removal measurements. However, Eco-QSAR or ECOSAR assessment should be regarded as supporting environmental-risk evidence rather than a direct indicator of TRL advancement. In addition, the reviewed studies employing ECOSAR generally did not report quantitative model-validation metrics or prediction accuracy. Therefore, predicted toxicity values should be interpreted as estimates requiring experimental confirmation rather than as substitutes for comprehensive ecotoxicological testing.

The transition from TRL 4 to TRL 5 therefore represents a major development gap for NGBN photocatalytic systems. At TRL 5, the technology must be validated under conditions that more closely represent its intended application. For wastewater treatment, the applicable criteria include: (i) validation using real or representative industrial/municipal wastewater; (ii) assessment of competing ions, natural organic matter (NOM), turbidity, suspended solids, and other matrix effects; (iii) validation under realistic and variable irradiation conditions; (iv) operation in continuous or semi-continuous reactor configurations; (v) extended assessment of catalyst stability and reusability; (vi) effective catalyst recovery and assessment of nanoparticle or metal-ion leaching; and (vii) more comprehensive assessment of degradation products, mineralization, and toxicity. The progression toward TRL 6–7 requires further engineering and operational validation. At TRL 6, the NGBN photocatalytic system should be demonstrated using an engineering-scale or pilot-scale reactor in a relevant environment, preferably with real wastewater under continuous or semi-continuous operation. At this stage, treatment capacity, hydraulic performance, catalyst recovery, irradiation efficiency, energy consumption, and sustained treatment performance should be established. TRL 7 would subsequently require demonstration of a full-scale-representative system under realistic industrial or municipal wastewater conditions, including variations in wastewater composition and operating conditions over an extended period. Accordingly, laboratory degradation efficiency alone would no longer be sufficient to demonstrate advancement at these levels. The gap summary presented in Table 3 further demonstrates that the principal limitation is not the ability of NGBNs to degrade dyes under laboratory conditions, but the lack of evidence demonstrating that this performance can be maintained under realistic wastewater-treatment conditions. In particular, real-water validation, continuous-flow operation, catalyst recovery, long-term stability, comprehensive environmental safety assessment, and techno-economic evaluation represent the major milestones separating the current TRL 3–4 systems from TRL 5–7 technologies. Thus, the next stage of development should focus on progressively increasing the complexity and scale of validation rather than simply improving degradation percentages under increasingly optimized laboratory conditions.^102–106^

**Table 3:** Gaps between the current status and required milestones for the industrial deployment of NGBN-based photocatalytic systems

Indicator	Current evidence from NGBN literature	Industrial target/required industrial milestone	Gap
NGBN reuse	3–7 cycles of reusability have been well reported and established	Long-term operation (up to 100 cyclic reuse)	>7 cycles not adequately demonstrated
Treatment mode	Batch	Demonstration of continuous operation	Continuous operation not demonstrated
Wastewater	Synthetic dye solutions	Real industrial effluent	Real-matrix validation limited
Scale	Laboratory	Pilot/industrial	Scale-up factor not established
TOC/COD	Reported, but very limited	Consistent quantification of TOC/COD removal	Major gap related to treatment efficiency
Energy demand	Not reported/rarely quantified	Quantification of energy demand (kWh m^−3^)	Critical data gap important for overall assessment
Treatment cost	Not reported/rarely quantified	Quantification of treatment cost (cost m^−3^)	Critical data gap important for overall assessment
Toxicity	Limited acute/ECOSAR studies	Comprehensive acute + chronic assessment	Major toxicological data gap
Real-world fouling/matrix effects	Not reported/rarely quantified	Long-term evaluation under variable wastewater composition	Operational validation gap

Beyond TRL 7, the technology enters the qualification and deployment stages. TRL 8 requires qualification of the complete treatment system through extended operational validation, techno-economic and life-cycle assessment, environmental and toxicological evaluation, and regulatory consideration, whereas TRL 9 represents reliable routine commercial operation with demonstrated technical, economic, environmental, and regulatory viability. Based on the criteria applied in this review, no NGBN-based photocatalytic dye-treatment system has yet demonstrated sufficient evidence to be assigned to TRL 8–9. Thus, the current literature remains primarily focused on laboratory validation and early-stage process development, with progression toward higher TRLs requiring successful fulfilment of the technical and operational milestones defined above.

Overall, the adapted TRL assessment indicates that NGBN photocatalytic systems remain predominantly at TRL 3–4, with limited evidence approaching TRL 5. Their current strength lies in well-established laboratory proof of concept and promising photocatalytic performance, whereas their principal gaps are summarized in Table 3. Addressing these gaps through staged validation from realistic laboratory matrices to continuous-flow systems and subsequently to pilot- and field-scale demonstration will be essential for translating NGBN photocatalysts from promising laboratory materials into viable technologies for sustainable wastewater treatment.

## 9. Challenges and environmental threats

One of the major challenges associated with NGBNs is their limited thermal stability. Natural gums generally undergo thermal degradation at relatively low temperatures and possess limited thermal strength compared with conventional synthetic polymeric matrices. Consequently, NGBNs may be vulnerable to structural breakdown under harsh or high-temperature photocatalytic conditions, potentially compromising their long-term stability and altering the surface chemistry and photocatalytic activity of the embedded nanoparticles. This limitation may restrict their application in processes involving elevated operating temperatures.

In addition to their limited thermal stability, the inherently variable composition of natural gums presents a significant challenge to the reproducibility of NGBNs. Their molecular weight, degree of branching, functional-group composition, and impurity content can vary depending on the plant source, geographical and climatic conditions, harvesting season, and extraction or purification method. Such variations may influence nanoparticle loading, dispersion, matrix structure, and ultimately the photocatalytic performance of the resulting NGBNs. Therefore, inadequate control of the raw material may lead to batch-to-batch inconsistencies and pose challenges for reproducibility, scale-up, and commercialization. Furthermore, the hydrophilic nature of many natural gums can result in considerable swelling or partial dissolution when NGBNs are exposed to aqueous environments for prolonged periods. Excessive swelling may alter the structure of the gum matrix and weaken its interaction with the embedded nanoparticles. In severe cases, this may facilitate gradual nanoparticle or metal-ion leaching during repeated photocatalytic cycles, thereby reducing the catalyst stability and potentially introducing additional environmental threats.

Beyond the intrinsic properties of the gum matrix, the presence of natural impurities and constituents of actual wastewater may affect the NGBN performance. Natural gums, particularly those obtained from waste or minimally processed plant materials, may contain residual proteins, minerals, organic compounds, or microbial contaminants that can influence photocatalytic reactions or promote undesirable side reactions. Moreover, real wastewater commonly contains natural organic matter, chloride, bicarbonate, and other inorganic ions that may scavenge reactive species such as hydroxyl radicals or compete for active sites on the photocatalyst. Consequently, the photocatalytic efficiency observed in laboratory-grade water can be challenging to replicate in complex wastewater matrices. Moreover, the limited availability of specific regulatory frameworks governing the environmental release, use, and end-of-life management of natural gum-based nanocomposite photocatalysts remains a challenge for their large-scale implementation. Although natural gums are generally considered biodegradable materials, the incorporation of metal or metal oxide nanoparticles introduces additional considerations regarding nanoparticle release, persistence, and potential ecological effects. The absence of standardized protocols for assessing these risks may complicate environmental certification, regulatory approval, commercialization, and scale-up.

Finally, an important environmental concern is the potential formation of toxic degradation intermediates during photocatalytic treatment. Although NGBNs can effectively promote the degradation of dye pollutants, the disappearance of the parent dye does not necessarily indicate complete mineralization or detoxification. Incomplete degradation may produce transformation products that are more persistent or toxic than the original pollutant, thereby creating a potential source of secondary pollution.^35,76^ This concern is particularly important, given that some degradation intermediates identified in studies involving NGBNs have exhibited toxicity.

## 10. Innovative pathways, emerging opportunities, and future perspectives

Considering the progress made so far on NGBNs for the photocatalytic degradation of dye pollutants, there are several innovative pathways and emerging opportunities (Fig. 12) that can serve as future research hotspots to help advance the field.

**Fig. 12:**
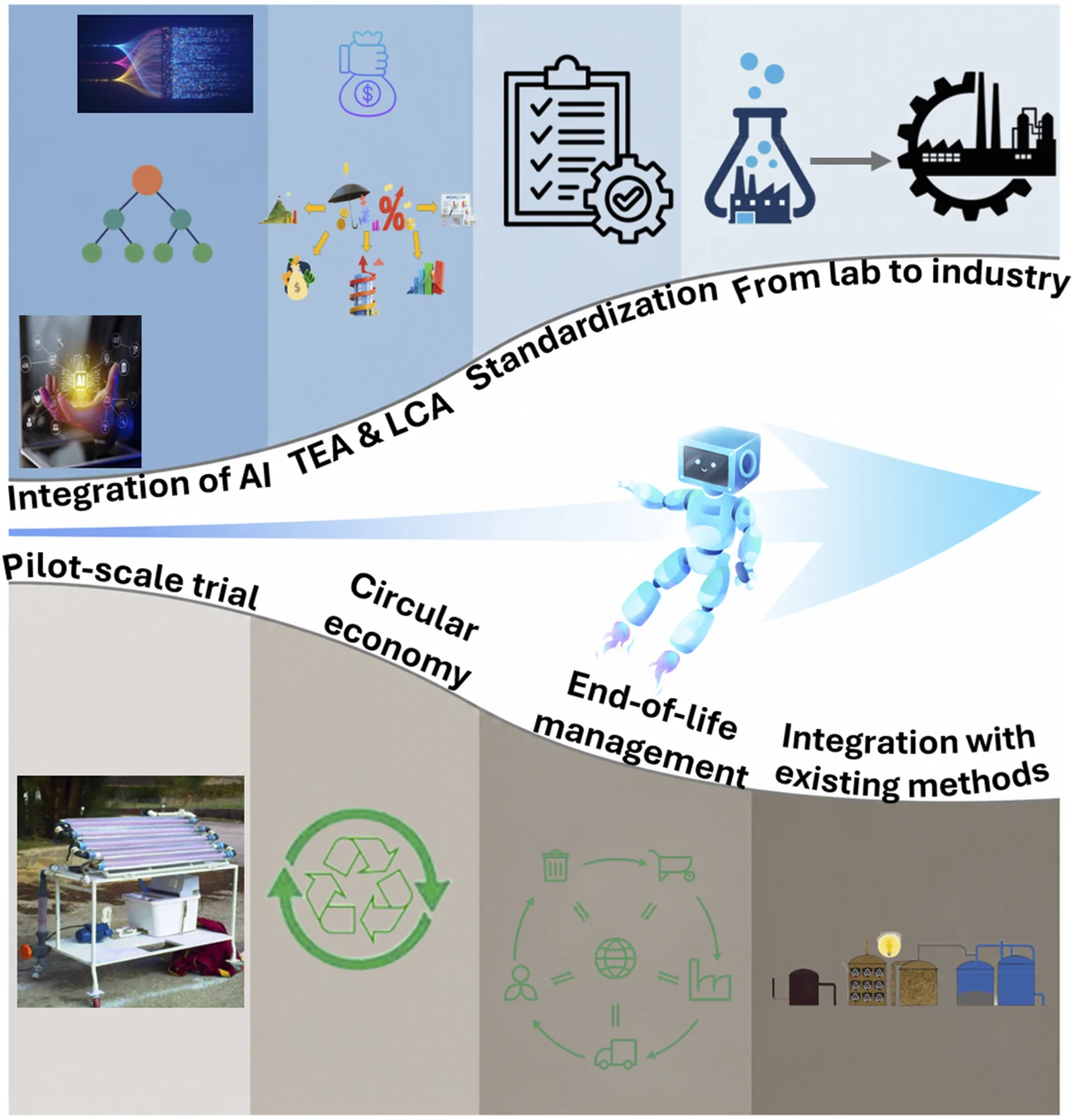
Innovative pathways and future perspectives. Adapted with permission from ref. 114. Copyright, John Wiley and Sons, 2025.

### 10.1. From lab to industry

Despite the remarkable progress achieved in the development of NGBNs, their application remains largely confined to laboratory-scale investigations employing synthetic dye solutions under controlled experimental conditions. While these studies have provided valuable insights into photocatalytic efficiency, degradation kinetics, and reaction mechanisms, they rarely replicate the complexity of real industrial wastewater. Consequently, the impressive degradation efficiencies frequently reported under laboratory conditions may not accurately reflect catalyst performance during practical wastewater treatment.

Industrial effluents generated by textile, pharmaceutical, leather, paper, printing, and municipal wastewater treatment facilities contain complex mixtures of contaminants beyond target dye molecules. These wastewater streams typically comprise high concentrations of dissolved inorganic salts, competing cations and anions, natural organic matter, surfactants, pharmaceuticals, suspended solids, heavy metals, oils, and fluctuating pH values. Such constituents can compete with dye molecules for active adsorption sites, reduce light penetration through increased turbidity, poison photocatalytically active surfaces, alter charge-transfer pathways, or scavenge reactive oxygen species, thereby substantially diminishing photocatalytic performance. Furthermore, catalyst fouling, mechanical degradation, nanoparticle leaching, and gradual deactivation during prolonged operation remain largely unexplored for NGBN systems.

Bridging the gap between laboratory-scale success and industrial application, therefore, represents one of the most important future research priorities. Future investigations should move beyond batch experiments and systematically evaluate NGBNs using authentic industrial wastewater under realistic environmental and operational conditions. Emphasis should be placed on understanding the influence of high dye concentrations, complex ionic backgrounds, seasonal variations in wastewater composition, hydraulic conditions, catalyst ageing, and repeated regeneration cycles on photocatalytic efficiency, mineralization performance, structural integrity, long-term durability, and catalyst reusability. Such studies should also assess the degradation of mixed-pollutant systems containing dyes together with pharmaceuticals, heavy metals, surfactants, and other emerging contaminants to better simulate real wastewater matrices.

Equally important is the transition from bench-scale reactors to pilot-scale and ultimately industrial-scale photocatalytic systems. Future research should focus on the design, optimization, and validation of continuous-flow photoreactors, immobilized photocatalyst systems, membrane-assisted photocatalytic reactors, slurry reactors with efficient catalyst recovery, and solar-driven treatment units capable of processing large wastewater volumes with minimal catalyst loss and reduced energy consumption. Reactor engineering parameters, including hydraulic retention time, catalyst loading, light distribution, photon utilization efficiency, mass transfer, mixing characteristics, residence time distribution, and hydraulic stability, should be systematically optimized to maximize treatment performance while minimizing operational costs.

Beyond validating pollutant removal efficiency, future investigations should comprehensively evaluate engineering performance indicators that determine the practical feasibility of industrial deployment. These include treatment capacity (m^3^ d^−1^), pollutant degradation kinetics under continuously fluctuating influent compositions, catalyst lifetime, regeneration efficiency, operational stability, and process robustness during prolonged operation. For continuous-flow systems operating under natural or simulated solar irradiation, additional performance metrics such as specific energy consumption (kWh m^−3^ of treated wastewater), space-time yield, quantum efficiency, photocatalyst productivity, solar utilization efficiency, and catalyst recovery efficiency should be routinely reported to facilitate meaningful comparisons among different reactor designs and operating conditions. Moreover, comprehensive mass balance analyses, total organic carbon (TOC) removal, chemical oxygen demand (COD) reduction, and identification of degradation intermediates should accompany photocatalytic studies to confirm complete mineralization rather than simple decolorization and to evaluate the potential formation of toxic transformation products. In parallel with reactor optimization, long-term pilot-scale demonstrations should be undertaken to evaluate operational robustness, catalyst regeneration, maintenance requirements, fouling behaviour, cleaning protocols, catalyst replacement intervals, and overall process reliability under continuous operation. Such investigations will generate the engineering, environmental, and economic data required for process scale-up, facilitate technology validation under realistic operating conditions, and accelerate the commercialization of NGBN-based photocatalytic technologies.

### 10.2. Integration with the existing water treatment infrastructure

Although NGBNs have demonstrated remarkable photocatalytic performance for dye degradation under laboratory conditions, their successful commercialization will depend not only on material performance but also on their compatibility with existing wastewater treatment infrastructure. Replacing conventional treatment plants with entirely new photocatalytic systems would require substantial capital investment and operational modifications, making large-scale implementation economically challenging. Consequently, future research should focus on developing NGBN-based technologies that can be readily integrated into existing treatment trains, thereby facilitating gradual industrial adoption while minimizing infrastructure costs.

From our own perspective, rather than functioning as standalone treatment technologies, NGBNs should be designed to complement conventional physical, chemical, and biological treatment processes. For example, photocatalytic reactors could be incorporated as tertiary treatment units following biological treatment to remove residual dye molecules, pharmaceutical contaminants, endocrine-disrupting compounds, and other refractory organic pollutants that are resistant to conventional degradation processes. Likewise, coupling NGBNs with membrane filtration systems could simultaneously achieve pollutant degradation and membrane self-cleaning, thereby mitigating membrane fouling and extending membrane service life. Integration with adsorption technologies, coagulation–flocculation, advanced oxidation processes (AOPs), electrochemical oxidation, ozonation, constructed wetlands, and activated carbon filtration may also generate synergistic effects that improve the overall treatment efficiency while reducing chemical consumption and operational costs.

Future studies should also investigate hybrid photocatalytic systems that combine NGBNs with complementary technologies capable of enhancing charge separation, light utilization, and pollutant mineralization. Examples include photoelectrocatalysis, photocatalytic membrane reactors, photocatalytic-biological treatment systems, solar-assisted advanced oxidation processes, and photocatalytic oxidation coupled with microbial degradation. Such integrated systems could exploit the strengths of multiple treatment technologies while overcoming the limitations associated with individual processes, including incomplete mineralization, catalyst recovery, high energy consumption, and limited treatment capacity.

From an engineering perspective, emphasis should be placed on developing modular photocatalytic units that can be retrofitted into existing wastewater treatment plants without major modifications to process infrastructure. Immobilized NGBNs supported on ceramic substrates, polymeric membranes, glass beads, stainless-steel meshes, activated carbon, or monolithic supports would facilitate catalyst recovery, reduce nanoparticle loss, and enable continuous operation within existing treatment facilities. Furthermore, solar-driven photocatalytic modules should be designed to integrate with existing open-channel reactors, maturation ponds, and tertiary treatment systems to reduce electricity consumption while maximizing renewable energy utilization.

Summarily, the successful translation of NGBNs from laboratory-scale innovation to practical wastewater treatment is unlikely to depend on replacing conventional technologies but rather on strategically integrating photocatalytic nanocomposites into the existing treatment infrastructure. Developing modular, hybrid, and easily retrofittable NGBN-based photocatalytic systems will significantly improve the prospects for industrial adoption, reduce implementation costs, and accelerate the deployment of sustainable photocatalytic technologies for large-scale remediation of dye-contaminated wastewater.

### 10.3. Sustainable end-of-life management and circular economy strategies

As NGBNs move closer to practical deployment, greater attention should be devoted to the environmental management of spent photocatalysts. Repeated photocatalytic cycles may result in the accumulation of degradation intermediates, adsorbed pollutants, dissolved metal ions, and structural degradation products that could pose secondary environmental risks if discarded without appropriate treatment. Consequently, catalyst disposal should be considered an integral component of future photocatalytic system design. Rather than simple disposal, future research should investigate sustainable regeneration, recycling, and value-added repurposing strategies. One promising approach involves converting exhausted NGBNs into functional carbon-based materials through controlled thermal treatment like torrefaction, pyrolysis, or hydrothermal treatment. Owing to the intrinsic carbon-rich nature of natural gums, spent photocatalysts may serve as precursors for porous conductive carbons applicable in supercapacitors, battery electrodes, electrocatalysts, or electrochemical sensors. Such circular-economy strategies would simultaneously reduce waste generation, recover valuable materials, minimize environmental impacts, and improve the overall economic sustainability of NGBN technologies. Life-cycle assessment studies should also be incorporated to quantify environmental benefits throughout catalyst production, utilization, regeneration, and final disposal.

### 10.4. Techno-economic analysis

Although numerous studies have demonstrated the photocatalytic performance of NGBNs, relatively little attention has been devoted to their economic feasibility and commercial viability. Most investigations emphasize degradation efficiency while overlooking critical factors such as raw material costs, synthesis complexity, energy consumption, reactor configuration, catalyst regeneration, operational expenses, and maintenance requirements. Without comprehensive economic evaluation, it remains difficult to determine whether NGBNs can compete with existing wastewater treatment technologies.

Future research should therefore incorporate detailed techno-economic analyses (TEA) alongside photocatalytic performance evaluation. Such assessments should quantify production costs, catalyst lifetime, regeneration efficiency, energy requirements, reactor productivity, cost per cubic meter of treated water, and overall process scalability. Integrating TEA with life-cycle assessment (LCA) will provide a holistic evaluation of both environmental and economic sustainability. These analyses will assist environmental engineers, wastewater treatment facilities, industrial stakeholders, technology developers, investors, and policymakers in determining the practical feasibility and commercialization potential of NGBN-based photocatalytic systems.

### 10.5. Standardization and regulatory assessment

Future investigations should adopt standardized testing procedures to improve reproducibility and facilitate objective performance benchmarking. In addition, greater attention should be directed toward environmental safety and regulatory compliance. Although natural gums are inherently biodegradable and environmentally benign, the incorporation of metal and metal oxide nanoparticles may introduce potential ecotoxicological concerns through nanoparticle leaching, bioaccumulation, or long-term environmental persistence. Comprehensive assessments of catalyst toxicity, nanoparticle release, environmental fate, and ecological impacts should therefore accompany future photocatalytic investigations. Furthermore, alignment with internationally recognized environmental and materials-testing standards will facilitate regulatory approval and accelerate the transition of NGBNs from laboratory-scale research to practical wastewater treatment technologies.

### 10.6. Integration of artificial intelligence (AI)

The integration of AI, machine learning (ML), and materials informatics represents a promising frontier for accelerating the development of NGBN photocatalysts.^115–117^ AI-assisted photocatalysis has considerable potential for wastewater treatment and environmental remediation because it can enhance catalyst design, performance prediction, and process optimization while substantially reducing the development time, experimental effort, and associated costs. Despite the growing application of AI in photocatalyst discovery, reaction optimization, and environmental engineering, its application to natural gum-based nanocomposite systems remains largely unexplored. Given the extensive diversity of natural gums, semiconductor materials, metal and metal oxide nanoparticles, dopants, synthesis routes, and operational parameters, conventional trial-and-error experimentation is both time-consuming and resource-intensive. Thus, integrating AI-driven methodologies into NGBN research presents an unprecedented opportunity to accelerate the discovery of highly efficient, sustainable, and application-oriented photocatalysts.

Future studies should exploit AI and ML approaches to establish predictive relationships among material composition, synthesis conditions, physicochemical characteristics, and photocatalytic performance. Machine learning algorithms including artificial neural networks (ANNs), random forests (RFs), support vector machines (SVMs), gradient boosting methods, Gaussian process regression, Bayesian optimization and others presented in Fig. 13 can be trained using existing photocatalytic datasets to predict critical material properties such as band-gap energy, crystallite size, specific surface area, adsorption affinity, charge-carrier lifetime, degradation efficiency, apparent reaction rate constants, mineralization efficiency, catalyst stability, and reusability. Furthermore, these predictive models could estimate degradation intermediates and their potential toxicities, thereby supporting the development of safer and more environmentally benign photocatalytic systems. By identifying optimal combinations of natural gums, nanoparticles, dopants, and synthesis parameters before experimental validation, AI-assisted models can substantially reduce experimental workload while improving the research efficiency and accelerating materials discovery.

**Fig. 13:**
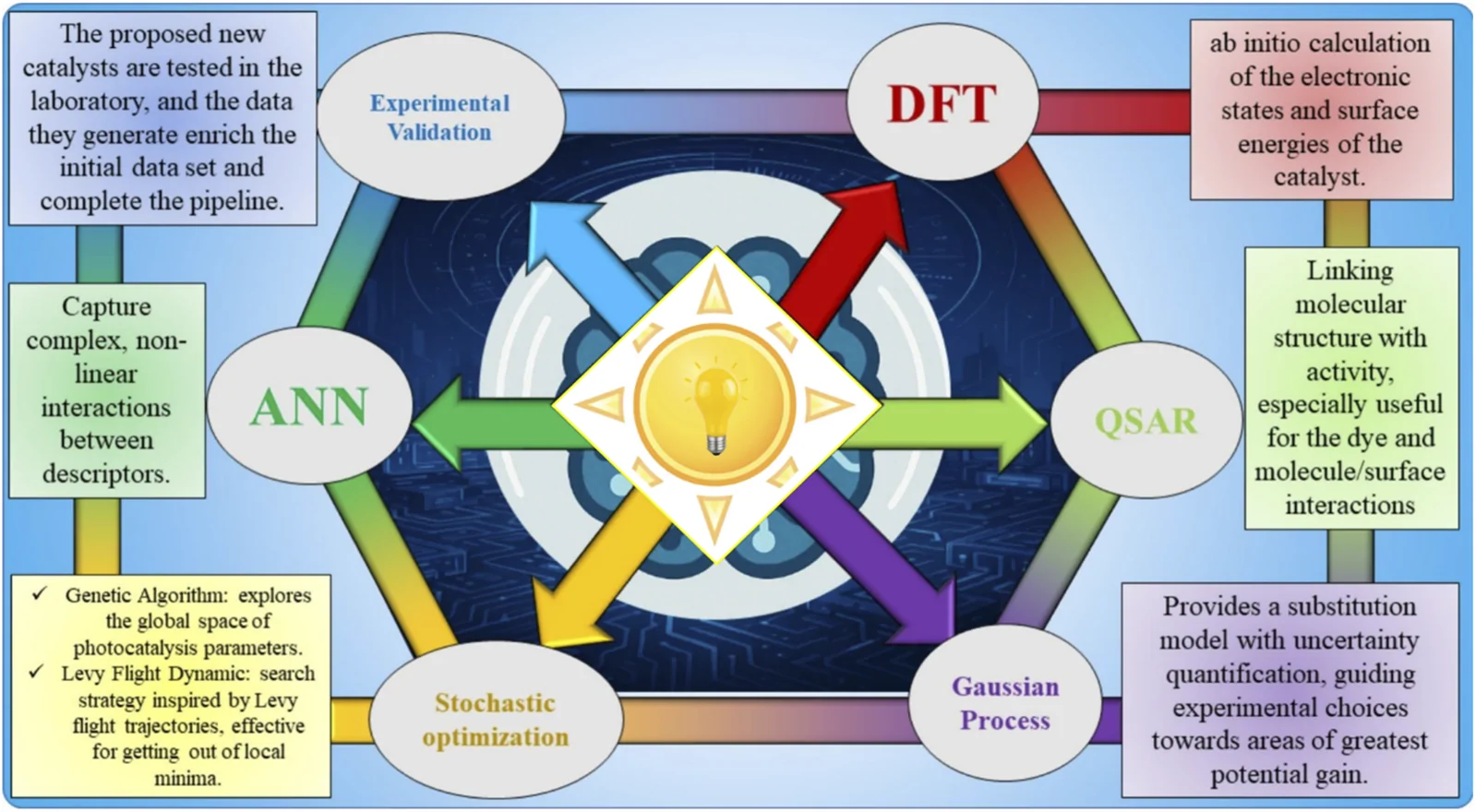
AI tools for optimization of dye photocatalysis. Reproduced with permission from ref. 117. Copyright, Elsevier B.V., 2025.

Beyond catalyst design, AI offers significant opportunities for optimizing photocatalytic reaction conditions. ML models trained on experimental datasets can predict the influence of variables such as catalyst dosage, solution pH, initial dye concentration, irradiation wavelength, light intensity, reaction time, temperature, oxidant concentration, and hydraulic retention time on the degradation efficiency and reaction kinetics. Such predictive capabilities would enable researchers to identify optimal operating conditions without exhaustive experimental screening. Emerging deep-learning frameworks developed for photocatalytic materials discovery, together with attention-based neural networks and transfer-learning approaches, further demonstrate the potential of AI to accurately predict photocatalytic performance under diverse experimental conditions, providing a powerful foundation for extending these methodologies to NGBN systems.

In addition to predictive modeling, advanced computational approaches should be integrated with experimental investigations to achieve a deeper mechanistic understanding of NGBN photocatalysis. Density functional theory (DFT), molecular dynamics (MD) simulations, and other atomistic modeling techniques can provide valuable insights into dye adsorption mechanisms, electronic band structures, charge distribution, oxygen-vacancy formation, heterojunction interfaces, electron transport pathways, charge-carrier dynamics, and reactive oxygen species generation. These computational tools can also elucidate how the functional groups present in natural gums influence nanoparticle stabilization, interfacial charge transfer, pollutant adsorption, and photocatalytic reaction pathways. The combination of AI-assisted data analysis with first-principles calculations would enable rational, mechanism-guided design of NGBNs possessing improved activity, stability, selectivity, and visible-light responsiveness.

It is also recommended that the role of AI should not be limited to materials discovery but should extend throughout the entire photocatalytic treatment process. Future photocatalytic reactors could integrate AI-enabled monitoring systems with real-time sensors capable of continuously measuring operational parameters such as influent dye concentration, pH, turbidity, dissolved oxygen, catalyst loading, irradiation intensity, flow rate, and hydraulic retention time. A typical example of the structure of a standard ANN that can be employed by future researchers to estimate the dye degradation percentage using NGBNs from input parameters such as light intensity, reaction time, temperature, pH, initial pollutant concentration, and catalyst dosage is illustrated in Fig. 14.^115,116^ This kind of ANN and AI algorithms could dynamically adjust these parameters during operation to maximize the degradation efficiency, improve catalyst utilization, minimize energy consumption, and maintain stable treatment performance despite fluctuations in wastewater composition. Such intelligent process-control systems would also enable continuous performance monitoring, predictive maintenance, early detection of catalyst deactivation or fouling, and automated operational optimization in pilot-scale and industrial-scale wastewater treatment facilities.

**Fig. 14:**
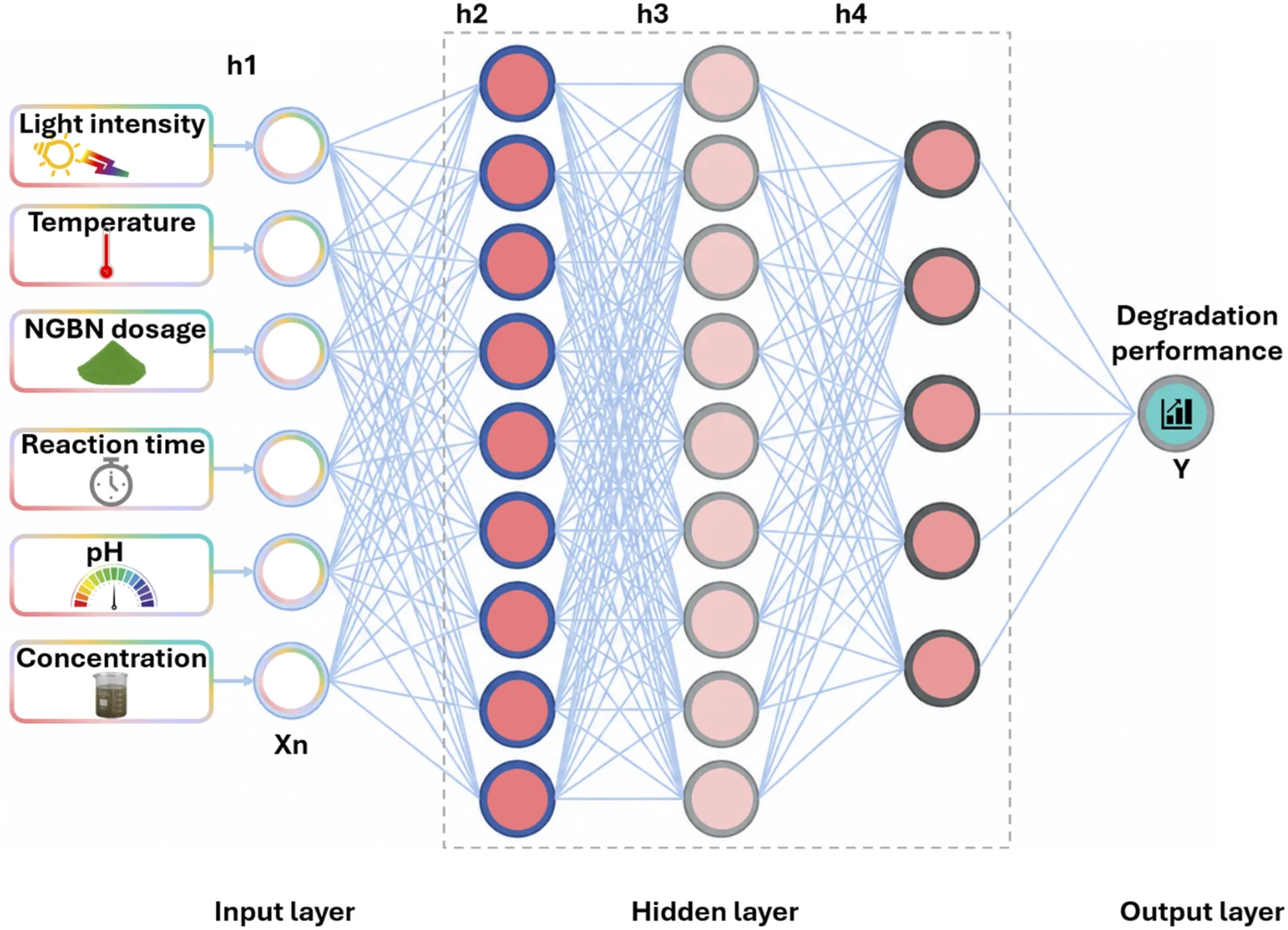
Example of an ANN model for predicting the efficiency of NGBNs for dye photocatalytic degradation based on experimental inputs. Adapted from ref. 116, which is open access and permits unrestricted use of materials under the terms of Creative Commons Attribution (CC-BY).

Ultimately, the convergence of AI, machine learning, high-throughput experimentation, computational chemistry, and intelligent reactor engineering has the potential to transform the development of NGBN photocatalysts from conventional empirical experimentation to data-driven materials innovation. Such an integrated digital framework would accelerate the discovery of next-generation photocatalysts, facilitate laboratory-to-industry translation, reduce research and operational costs, and support the deployment of efficient, scalable, and sustainable photocatalytic technologies for the remediation of dye-contaminated wastewater.

## 11. Conclusion

In this study, the current approach to the use of various natural gum-based nanomaterials for the photocatalytic degradation of dye pollutants was reviewed. Several key findings, advances, and trends were derived. First, it was found that the incorporation of natural gum specifically reduces the band gap value and improves the Schottky barrier of NGBNs, which then enhances e^−^/h^+^ separation and prolongs the e^−^/h^+^ recombination duration for enriched photocatalytic degradation activity. It was also discovered that the presence of natural gums imparts NGBN photocatalysts with an abundance of oxygenated functional groups (OH, COOH, and OCH_3_) and active sites, which foster effective charge transfer and radical generation (especially ˙OH) for enhanced photocatalytic degradation efficiency. Notably, the study findings revealed that various NGBNs show consistent trends of improved degradation efficiency >75% in most cases at a residence time of 1–760 minutes, an NGBN dose of 0.5–1000 mg, and a light intensity of 8–500 W. Moreover, for best practices, an average NGBN dose of ∼20–150 mg and a residence time of 124 minutes are recommended. Recent advances discovered include the use of natural sunlight for energy efficiency and the development of hybrid/assisted photocatalytic degradation operation using H_2_O_2_, which enhances radical generation as well as the photocatalytic degradation efficiency. The reusability and photostability studies also show that NGBNs can be reused 3–7 times while still delivering ≥85% efficiency without compromising the original architectural morphology or structure, which were largely found to be spherical, mesoporous, and crystalline in nature. This excellent reusability and photostability showcase the industrial potential of NGBNs. Furthermore, water and ethanol are the commonly employed regenerating/eluting agents, and this revealed the eco-friendliness and sustainability of this degradation technology. In addition, Eco-QSAR assessments suggest that some NGBNs exhibit low predicted acute toxicity; however, some dye degradation intermediates were predicted to be toxic. This highlights that photocatalytic degradation does not necessarily result in complete detoxification and that further studies are needed to establish the long-term environmental safety of NGBNs. Additionally, findings show that NGBNs' photocatalytic degradation system for dye pollutants is currently at TRL 3–4. Thus, future researchers need to concentrate more effort on conducting a comprehensive study on real effluent and pilot-scale trials in order to move this remediation process to higher TRL and further establish the overall effectiveness and eco-economic benefits. In the end, this study helped identify the strengths, weaknesses, opportunities, and threats that can serve as future directions in the research area. The generated results discussed in this review work suggested that the studied natural gum-based nanomaterials are viable, and could be envisaged for the effective treatment of dye-polluted wastewater.

## Author contributions

Stephen Sunday Emmanuel: conceptualization, methodology, writing – original draft preparation, supervision, validation, project administration, writing – reviewing and editing. Mustapha Omenesa Idris: writing – original draft preparation, validation, writing – reviewing and editing. Ademidun Adeola Adesibikan: writing – original draft preparation, validation, writing – reviewing and editing. Hamza Badamasi: writing – original draft preparation, validation, writing – reviewing and editing. Olumide James Oluwole: writing – original draft preparation, validation, writing – reviewing and editing.

## Conflicts of interest

The authors do not have any financial or non-financial conflicts of interest to declare.

## Data Availability

Data sharing is not applicable to this article as no datasets were generated or analyzed during the current study.
